# The Potential Emergence of “Education as Mental Health Therapy” as a Feasible Form of Teacher-Delivered Child Mental Health Care in a Low and Middle Income Country: A Mixed Methods Pragmatic Pilot Study

**DOI:** 10.3389/fpsyt.2021.790536

**Published:** 2021-12-16

**Authors:** Christina M. Cruz, Priscilla Giri, Juliana L. Vanderburg, Peter Ferrarone, Surekha Bhattarai, Aileen A. Giardina, Bradley N. Gaynes, Karen Hampanda, Molly M. Lamb, Michael Matergia

**Affiliations:** ^1^Department of Psychiatry, University of North Carolina at Chapel Hill School of Medicine, Chapel Hill, NC, United States; ^2^School Psychology Program, University of North Carolina at Chapel Hill School of Education, Chapel Hill, NC, United States; ^3^Darjeeling Ladenla Road Prerna, Darjeeling, India; ^4^Department of Global Health and Development, London School of Hygiene and Tropical Medicine, London, United Kingdom; ^5^Broadleaf Health & Education Alliance, Stroudsburg, PA, United States; ^6^Department of Epidemiology, Gillings School of Global Public Health, University of North Carolina at Chapel Hill, Chapel Hill, NC, United States; ^7^Center for Global Health, Colorado School of Public Health, Aurora, CO, United States; ^8^Department of Obstetrics and Gynecology, University of Colorado Anschutz Medical Campus, Aurora, CO, United States; ^9^Department of Epidemiology, Colorado School of Public Health, Aurora, CO, United States

**Keywords:** teacher, task-shifting, child mental health, feasibility, fidelity, global mental health, school mental health, education as mental health therapy

## Abstract

**Objective:** We assessed task-shifting children's mental health care to teachers as a potential approach to improving access to child mental health care.

**Methods:** In Darjeeling, India, we conducted a single-arm, mixed-methods feasibility study with 19 teachers and 36 children in five rural primary schools to determine whether teachers can deliver transdiagnostic mental health care to select children-in-need with fidelity to protocol, to assess which therapeutic options teachers chose to use within the protocol, and to evaluate for a potential signal of efficacy.

**Results:** Participation rates for intervention activities were >80%. A majority of teachers met or exceeded quality benchmarks for all intervention activities. Teachers chose to deliver teacher-centric techniques, i.e., techniques that only teachers could deliver given their role in the child's life, 80% of the time. Children improved in mental health score percentiles on the Achenbach Teacher Report Form. Key facilitators included the flexibility to adapt intervention activities to their needs, while identified barriers included limited time for care delivery.

**Conclusion:** Findings support the feasibility of task-shifting children's mental health care to classroom teachers in resource-limited schools. Fidelity to protocol appeared feasible, though the freedom to choose and adapt therapeutic techniques may also have enhanced feasibility. Surprisingly, teachers consistently chose to deliver teacher-centric therapeutic techniques that resulted in a potential signal of efficacy. This finding supports the potential emergence of “education as mental health therapy” (Ed-MH) as a new therapy modality. Continued investigation is required to test and refine strategies for involving teachers in the delivery of transdiagnostic mental health care.

## Introduction

Most of the 20% of children with mental health needs will remain unrecognized, unsupported, and affected throughout their lives (1, 2). Access to quality mental health care remains poor for most, particularly in low and middle income countries (LMICs) such as in India where <1% of affected children receive care (3). Innovative care models are urgently required to overcome this care gap (4, 5).

Task-shifting mental health care, in which professionals train and support non-accredited lay counselors to deliver therapy, is one widely used approach to close mental health care gaps in LMICs for adults and adolescents (6). Task-shifting indicated child mental health care, however, has yielded mixed results, in part as its provision requires knowledge of and experience with children's developing cognitive and emotion-regulation abilities (7).

With relevant experience in child development, teachers in LMICs are uniquely positioned to deliver indicated task-shifted children's mental health care given their consistent access to children (8, 9). However, few publications in LMICs study interventions that shift to teachers the task of delivering mental health care to select children-in-need (6, 7, 9), perhaps due to teachers expressing lack of (1) training to do so and (2) time and energy to take on counseling tasks above their primary duties (4, 8).

Yet, relevant literature studying teachers delivering other forms of mental health care, specifically mental health prevention and promotion for children and indicated care for adolescents, indicate that teachers in LMICs can feasibly deliver this care with fidelity (7, 10–15). Notably, these interventions are structured as whole-school or whole-class interventions with elements that fit into teacher workflows, folding an alternative structure in with more typical one-on-one sessions lay counselors conduct (6, 7, 10, 14, 15). Further, as implementation fidelity is considered imperative in any task-shifting model, studying teacher fidelity to any protocol is crucial in determining whether teachers can deliver indicated, task-shifted mental health care (16, 17). No published studies have assessed the feasibility of teachers delivering with fidelity indicated, task-shifted, alternatively structured mental health care. Such an evaluation may have important implications for the viability of teachers delivering care to children with mental health needs.

The purpose of this study was to determine the feasibility of teachers delivering “Tealeaf” with fidelity, where “Tealeaf” (*Teachers* *Leading the* *Frontlines - Mansik Swastha; Tealeaf – Mental Health; “Tealeaf”*) is a novel intervention our team developed in which teachers deliver evidence-based, indicated psychological care to their students in need in rural primary schools of Darjeeling, India. Departing from traditional lay counseling models where care is solely delivered in one-on-one, office-based sessions, Tealeaf is unique in that teachers use a transdiagnostic, non-manualized, evidence-based approach that they can customize, including using both one-on-one sessions and care tasks fitted into their primary teaching duties. To evaluate the feasibility of Tealeaf, in 2018, we used a convergent parallel mixed methods design. We hypothesized that, with training, support, and supervision, classroom teachers could deliver indicated mental health care, Tealeaf, with fidelity, and that such fidelity was feasible for teachers. We further hypothesized that teachers would primarily choose therapeutic techniques traditionally used by lay counselors in one-on-one sessions and then choose supplementary classroom-based techniques to reinforce the therapeutic work completed in one-on-one sessions. Secondary aims were (1) to explore potential impact on student mental health statuses and (2) to assess whether fidelity of intervention delivery was associated with changes in children's mental health status.

## Materials and Methods

### Setting

Darjeeling is a district in the State of West Bengal in the Himalayas of Northeast India. Within India, Darjeeling is considered a multi-ethnic, geographically-distinct space with several minority communities existing within and side-by-side with Nepali residents (18). Economic conditions in the predominant tea plantation and small-scale agricultural communities are poor, with daily wages for tea labor at 202 INR ($2.75 USD) (18).

Due to a perception of higher quality education and preference for English education, many families in rural Darjeeling send their children to low-cost private (LCP) schools (19). While Darjeeling estimates of LCP enrollment are not available, LCP enrollment is estimated at 30–50% of primary school students across India (18). LCP schools are primarily established by community members charging modest tuition to cater to a low-income population (19). As a result, LCP school teachers get paid minimally, 1,500–3,000 INR ($23–45 USD) monthly (18). No study has published the demographics of teachers in Darjeeling; one study that aggregates LCP and government teacher demographics across India shows that, compared to government school teachers, LCP teachers were more likely to be younger (29.61 vs. 40.28 years) and be college graduates (49 vs. 39%), and less likely to complete a teaching certificate (28 vs. 80%) (20).

### Participants

Seventeen LCP schools were approached; five were pragmatically selected. Inclusion criteria were set with the goal of reaching children in Darjeeling with poor access to care (19). Eligible schools could not be receiving government aid, could charge annual fees of 11,500 INR ($180 USD) or less, and were located in rural Darjeeling. Also, schools had to have four full-time teachers on faculty and enroll at least 50 students to increase the likelihood of accurate approximation of child mental health prevalence rates globally.

From these schools, 23 teachers were consented, 21 attended the Tealeaf 10-day training, and 19 teachers completed all study activities. Teachers were eligible if they had primary teaching responsibility for a primary grade level class (1–4) in an enrolled school, were 18 years or older, and were not suspected or convicted of child-related misconduct. Teachers were required to have >1 year of teaching experience to avoid confounding the feasibility of care delivery with learning how to teach. Teachers were not compensated for their participation in the study.

After teachers completed training, they used a study specific tool, the Behavior Type and Severity Tool (BTST; Supplementary Figure 1), to systematically capture their clinical impressions of each student in their class (ranging from 5 to 25 students). Tealeaf-trained teachers then each nominated two of their students in need of mental health care for their mental health services. The subject of a separate publication, teachers nominated children in need in this study with moderate accuracy, using their judgment and as aided by their impressions captured by the BTST (21, 22). The BTST as a stand-alone tool is weakly accurate in identifying students in need of mental health care as it was designed as a tool to aid teachers in their decision rather than as a definitive diagnostic tool; the moderate accuracy of teacher nomination largely stemmed instead from teacher judgment after completing training (21). Teachers were pragmatically limited to nominating two students each as, in earlier pilot testing, teachers voiced managing up to 2 students each feasibly given their education duties (21, 22).

The sole inclusion criteria for children (6–12 years old) was that they were in the class of an enrolled teacher. This inclusion criteria was set to be minimal to (1) to test teacher accuracy in nominating their students for mental health care and (2) allow children with any category of mental health difficulty and with any level of severity to be in the trial and receive care, as is the aim of Tealeaf. Tealeaf is designed to be resource-minimal given its LMIC setting and correspondingly leverages teachers for many care tasks, including selection of children for care. Thus, part of what was tested in this trial was teacher accuracy of nomination, as discussed above and the subject of a separate publication (21). Further, teachers are taught to deliver indicated care to students in need of that level of care. As a result, all levels of severity of symptoms were included; per study protocol and under the guidance of the principal investigator (CMC), though, children whose mental health difficulties endanger the safety of themselves or others are acutely referred to the local health system as is local custom (23, 24). Moreover, Tealeaf is designed to be transdiagnostic, as later described, such that teachers can deliver care to any child with any mental health struggle. Accordingly, all diagnostic categories of mental health symptoms were included.

### Intervention

Tealeaf is an intervention that task-shifts to teachers the delivery of evidence-based, indicated child mental health care. It is a non-manualized approach that teachers can customize, including fitting care tasks into their primary teaching duties. Further, Tealeaf is designed to be transdiagnostic as this approach to care applies “the same underlying principles across mental disorders, without tailoring the protocol to specific diagnoses,” allowing teachers to learn one set of therapeutic techniques and still provide care for any diagnosis (25). It was created in response to community needs identified through a lay field-worker-led school health program for primary schools in Darjeeling, India (19).

Tealeaf is implemented over a school year and involves six major components: training and supervision, nomination of students for care, behavior analysis, behavior plans, one-on-one interaction with students, and engaging caregivers. Basic functional behavioral assessments and Cognitive Behavior Play Therapy (CBPT) serve as core tenets of care. Teachers understand their students' mental health status and behavior through basic functional behavioral assessments. They then deliver care through the use of evidence-based therapy techniques, derived from CBPT, that are interwoven into a child's daily school schedule. CBPT was chosen as Tealeaf's core therapeutic modality as it provided teachers with practical and tangible therapeutic techniques based in Cognitive Behavior Therapy (CBT) that are cognitively accessible to primary-school-aged children using both talk and play forms of therapy (26). The efficacy of a play therapy medium for primary school students has been repeatedly validated while the efficacy of CBT has been extensively validated for older children, with evidence to support use in younger children (27). Table 1 breaks down Tealeaf into deliverable components.

**Table 1:** Core intervention components.

**Intervention component**	**Activity**	**Description**
Professional development & regular supervision	Training	PD: 10-day interactive training for teachers in identification of children with mental health needs, basic functional behavioral assessments and tenets of CBPT, particularly behavior activation, play, and cognitive restructuring Supervision: Twice monthly discussion with and/or observation of the teacher working with the child to provide concrete guidance and technique Case Reviews: Monthly case reviews are conducted with a team of local and international mental health experts.
Assessment	Behavior analysis	Observations of targeted students through a behavioral lens for key behaviors using the AABC Chart and the Themes of the AABC Chart. Supplemented by collateral from caregivers for further observations from student's home lives.
Tailored instruction; therapeutic interactions & skills practice	Behavior plan	A behavior plan (4Cs) incorporating CBPT and classroom-based therapeutic techniques that target school-specific behaviors (1:1 and during instructional time), to be used daily. Use of plan in student home is highly encouraged.
Therapeutic interactions & skills practice	1:1 student interaction	Per behavior plan students engage in 1:1 interactions with teacher during or outside of class. These interactions include CBPT and relationship-building activities.
Therapeutic interactions & skills practice	1:1 family interaction	With support and guidance from teachers, primary caregivers have roles in behavior analysis, implementation of behavior plans, development of positive parental relationships, and reinforcement of positive behaviors.

*PD, professional development; CBPT, Cognitive Behavior Play Therapy; AABC Chart, Activating Event, Automatic Thought and/or Feeling, Behavior, & Consequence Chart; 4Cs, Cause, Change, Connect, Cultivate; 1:1, one-on-one*.

A psychiatric social worker with expertise in youth mental health first delivers a 10-day training (see Supplementary Figure 2 for training outline). While the approach to care is transdiagnostic, a framework of behavior fitting into categories of anxiety, disruptive, or mood is taught to technically level for teachers the conceptualization of mental health behaviors and accompanying care, discussed further in a separate publication from this group of authors (21). Categories were named “nervous,” “disagreeable,” and “withdrawn,” respectively, after consulting with local experts and to refrain from further stigmatizing mental health.

Teachers then use a study specific tool, the BTST (Supplementary Figure 1), to systematically capture their clinical impressions of each student in their class. Using their judgment, aided by the BTST (as discussed above), they each choose two students to deliver care to, pragmatically limited, whom they believe have the highest mental health needs (21).

Thereafter, teachers analyze the behavior of each child they choose to deliver care to using 2 decision support tools, the Activating Event, Automatic Thought and/or Feeling, Behavior, & Consequence Chart (AABC Chart) and the Themes of the AABC Chart, that simplify and technically level behavior analysis (Supplementary Figures 3A,B). The AABC Chart is similar to an ABC Chart in CBT with the exception of the addition of “automatic thoughts and/or feelings.” This was added (1) to simplify the distinction between automatic thoughts and resulting feelings as teachers were considering them as one and the same in the pilot and (2) to set up their later work with students to identify the automatic thoughts and/or feelings associated with the observed behavior as part of cognitive restructuring. The Themes of the AABC chart was created as a decision support tool to further technically level the interpretation of the AABC Chart that teachers had expressed difficulty interpreting during pilot testing.

After behavior analysis, teachers then developed a targeted response using a behavior plan called the 4Cs Plan (Cause, Change, Connect, and Cultivate) (Supplementary Figure 3C). The 4Cs is akin to behavior plans teachers commonly use in high income countries (HICs) to manage a child's challenging behavior toward the goal of improved learning, but used in Tealeaf with the end goal of improving child mental health (28). An individualized behavior plan approach was chosen as the care implementation method (as opposed to manualized care) as it aligned with how teachers already adapted their teaching and interactions individually to students' needs (8). In the 4Cs, teachers select therapeutic techniques to deliver from (1) a menu of evidence-based therapeutic options to use for each category of behavior, (2) ones learned in training that were not listed on the menu of options, or (3) ones they adapted under the guidance of study staff (PG) (Supplementary Figure 4). In Tealeaf, the “dose” of care is each interaction between the teacher and student that is tweaked to be therapeutic and evidence-based, whether in the classroom or in one-on-one brief sessions. Such a dosing system deviates from traditional lay counselor task-shifted care where “doses” are measured as number and length of office-based, one-on-one therapy sessions.

**Figure 1:**
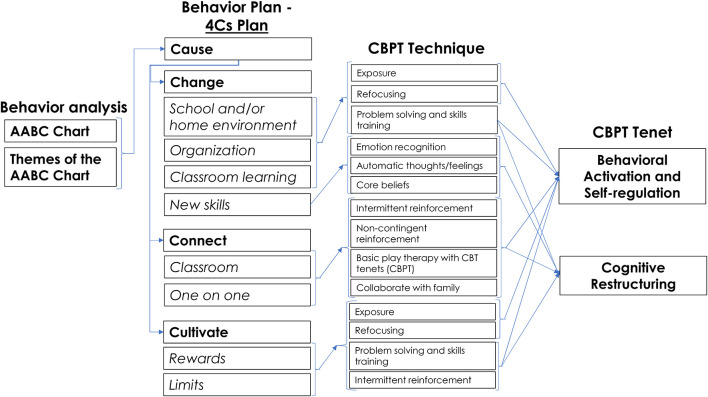
Mapping out CBPT tenets and techniques within Tealeaf.

CBPT techniques and tenets are infused throughout the 4Cs (Figure 1). As part of technically leveling CBPT, available techniques include ones that can fit within classroom activities or school schedules. To further technically level CBPT and increase potential feasibility, (1) behavioral activation and self-regulation and (2) cognitive restructuring are the chosen areas of therapeutic focus for teachers (Figure 1). Considered complementary in CBT and in neurocognitive systems, behavioral activation and self-regulation can be guided by teachers as it actually occurs, in contrast to an office-based therapist who is limited to planning and debriefing away from lived moments (8, 29, 30). Cognitive restructuring is incorporated through traditional means, such as the AABC Chart; it is also taught to be used in moments of struggle or success during which teachers are providing evidence to counter automatic thoughts/feelings and core beliefs in real time, similar to behavioral activation and self-regulation as above (8).

Following completion of the 4Cs, the remainder of the school year is dedicated to the development of therapeutic relationships and the delivery of therapeutic interactions and skills practice, with revisions to the 4Cs based on each child's progress. Teachers make changes to the school environment and engage with the child throughout the school day and in one-on-one (1:1) interactions. Real time interactions allow for immediate positive reinforcement of appropriate behaviors, as well as real time crisis management, processing of co-experienced behavioral struggles, and learning of social skills within the student's everyday setting. Rooted in each child's 4Cs Plan (and not manualized, as discussed above), teachers have the freedom to choose the content of their one-on-one interactions and sessions as well as how many, how long, and where their interactions and sessions will be. With the same level of freedom as the care-delivery, teachers also work with families to guide development of positive parental relationships and reinforcement of positive behaviors in the home. Throughout the year, teachers receive supervision through monthly site visits supplemented by as-needed telephone discussions by the psychiatric social worker with youth mental health expertise who also delivers their training to guide their care, leading to two supervision sessions monthly on average.

### Outcomes and Procedures

Fidelity was defined as the degree to which teachers implemented intervention activities as intended (16). Four elements of fidelity were assessed: (1) adherence, (2) exposure, (3) quality of delivery, and (4) participant responsiveness (31). To assess these elements of fidelity, we mapped quantitative indicators to the core components of the intervention (Table 2). Of note, evaluation of the training component is the subject of a separate manuscript (32).

**Table 2:** Intervention components with associated fidelity measures and elements.

**Intervention component**	**Measure**	**Fidelity elements**
Behavior analysis	Participation rate^a^	Exposure; Participant Responsiveness
	Time log	Exposure; Participant Responsiveness
	AABC observation tool^b^	Adherence; Quality of Delivery
Behavior plan	Participation rate^a^	Exposure; Participant Responsiveness
	Time log	Exposure; Participant Responsiveness
	4Cs observation tool^c^	Adherence; Quality of Delivery
1:1 student interaction	Participation rate^a^	Exposure; Participant Responsiveness
	Time log	Exposure; Participant Responsiveness
	Student observation tool^d^	Adherence; Quality of Delivery
1:1 family interaction	Participation rate^a^	Exposure; Participant Responsiveness
	Time log	Exposure; Participant Responsiveness
	Family observation tool^e^	Adherence; Quality of Delivery
Overall	Time log	Exposure; Participant Responsiveness

*1:1, one-on-one.*

*^a^Participation rates are the proportion of teachers observed as completing the intervention components.*

*^b^The AABC observation tool is a study-specific checklist used to evaluate teacher-completed AABC charts (a support tool to facilitate teachers in completing functional behavioral analysis).*

*^c^The 4Cs observation tool is a study-specific checklist used to evaluate teachers completed 4Cs Behavior Plans.*

*^d^The student observation tool is a study-specific rubric used to evaluate therapeutic interactions between the teacher and student.*

*^e^The family observation tool is a study-specific rubric used to evaluate therapeutic interactions between the teacher and student's caregiver(s)*.

Time logs and participation rates for intervention activities were used to assess exposure (the amount of the intervention delivered) and participant responsiveness (the extent to which teachers engaged in intervention activities). Teachers were requested to record the time spent and nature of interactions with the students (Supplementary Figure 5). A mean time investment of >30 min/week was chosen to indicate sufficient exposure and participant responsiveness, similar to other interventions in which lay counselors deliver task-shifted mental health care (6). Participation rates were defined as the proportion of teachers participating in core intervention activities, determined by direct observation by study personnel.

To assess adherence (whether teachers delivered the intervention as it was designed) and quality of delivery (the manner in which the teacher delivered the intervention), observation tools were developed for the four intervention activities (Supplementary Figure 6). These study-specific checklists and rubrics were developed by the research team, reviewed by a panel of experts, and field tested prior to finalization.

Teacher-completed AABC Charts and 4Cs Plans were rated using checklists that assessed understanding of theoretical concepts and application of key skills. Each item on the checklist was rated all (1), some (0.5), and none (0) and the results were averaged to yield a final score expressed as a percentage. Therapeutic interactions with the child and family were rated based on observations of counseling skills. Guided by rubrics, observers rated teachers from 1 to 5, 5 being of highest quality, at 0.5 intervals before determination of a final score.

We determined an *a priori* benchmark score of ≥60% for each observation tool to indicate that teachers delivered the intervention component as intended and with high quality. To set a high standard, 60% was chosen based on literature for rating fidelity when mental health professionals were demonstrating a new technique (33–35).

Observational assessments were conducted by the Research Administrator (PG). Multiple procedures were utilized to ensure reliability in ratings. The Research Administrator is a regional expert in child mental health with deep familiarity with the intervention. During 60 hours of training on the study-specific evaluation tools, she collaboratively scored a sub-sample of AABC Charts and 4Cs Plans with the PI (CMC) and reviewed observations of therapeutic interactions with the PI to norm scoring processes. She and the PI were blinded to the predetermined benchmarks.

Qualitative data were acquired in parallel to gain an in-depth understanding of feasibility. Semi-structured interviews (“interviews”) with 17 teachers were conducted in December 2018 (Supplementary Figure 7) and supplemented with intervention documentation (e.g., field notes and supervision notes). Interviews were recorded and conducted in Nepali; they were transcribed and translated into English by an outside agency. 4Cs Plans and field notes documenting changes to 4Cs Plans over time were also qualitatively evaluated to gain a descriptive understanding of the types of therapeutic measures teachers chose to implement.

To explore intervention impact on child mental health, students receiving care were assessed via the Achenbach Teacher Report Form (TRF) (36). Considered a gold standard, the TRF is standardized to capture teacher reports of child mental health challenges. Used globally in multicultural research, it has robust validity across LMICs, with evidence for partial support for factorial validity in the Indian context (37–39). Lower cutoffs for “clinical” and “borderline” designations than the TRF authors' published standards are thought to be more appropriate for the Indian context (37, 38). Still, no other teacher input form reviewed by a panel of experts was considered as locally applicable or as strongly validated for the local context as the TRF (37–39).

Based on teacher responses to 113 questions, several clinical scores are calculated on the TRF, with aggregate scores for Total Problem, Internalizing problems, Externalizing problems, Sluggish Cognitive Tempo, Obsessive Compulsive, and Stress Related, as well as subdomain scores for 8 empirically validated syndromes and 6 Diagnostic and Statistical Manual (DSM)—oriented scales. Raw scores are converted into T-scores, and a percentile score is obtained. Total problem, Externalizing, and Internalizing T-scores from 60 to 63 are considered “borderline” and ≥63 are “clinical.” The remaining aggregate scale scores and all subdomain T-scores from 65 to 69 are classified as “borderline” and ≥70 as “clinical.” Percentile scores <83 are considered “normal,” 83–90 “borderline,” and >90 “clinical” for Total Problem, Internalizing, and Externalizing scores. For the remaining aggregate scores and all subdomain scores, percentile scores <93 are considered “normal,” 93–97 “borderline,” and >97 “clinical.” For each child, TRFs were obtained pre-intervention (May), midpoint (September), and post-intervention (December). Each child was independently rated by two teachers: the primary observer was the teacher providing care and the secondary observer was another teacher not directly supporting the child but familiar with their behaviors and providing care to other students.

All participants provided written informed consent. For children, consent was obtained from a parent/guardian and children ≥7 years old provided verbal assent. The research protocol was approved by the University of North Carolina Institutional Review Board and a Darjeeling-based Ethics Committee. The study is registered with the Clinical Trials Registry-India (CTRI/2018/01/011471).

### Analysis

To study fidelity outcomes, we conducted a series of descriptive analyses where appropriate proportions meeting predefined benchmarks were computed (a priori analysis). To explore impact, we calculated mean percentile scores at each time point for each observer category (a priori analysis). A linear regression analysis was conducted to compare TRF percentile scores across the time period of observation with β representing the slope for the best-fitting line fitted to the three TRF scores in order (baseline, midpoint, endpoint), adjusted for gender (a priori analysis). Pearson's correlation coefficients were calculated to assess whether there was a statistically significant relationship between each aspect of fidelity (time logs and observation ratings for core activities) and children's mental health outcomes (TRF total problem score percentiles) (*post-hoc* analysis). All *P* values were 2-tailed and significance was set at *P* < 0.05. Analysis was completed in SAS version 9.4 (40).

Qualitative analyses were pursued with two aims. First, interviews were analyzed with the aim of qualitative description of the feasibility of teacher-delivered mental health care (a priori analysis) (41). Second, we aimed to capture teachers' choices of therapeutic techniques through qualitative description of the 4Cs and the research administrator's accompanying field notes documenting changes to the 4Cs throughout the year (*post-hoc* analysis). Two independent analysts coded all 4Cs Plans, field notes, and interview transcripts and converged on codes for each document. Coding and analysis was an iterative process of reading, coding, summarizing, and rereading. One codebook was created for 4Cs Plans and field note analysis and a second codebook for interview analysis. Using content analysis, coding began through a deductive coding method using an unconstrained matrix, allowing emergent codes to be added (42).

For interviews, codes were coalesced and a provisional set of themes and sub-themes were generated and reviewed, with important contrary opinions identified. Ultimately, a final set of common themes and sub-themes were identified alongside illustrative quotes. Results of the analysis were linked to common aspects of feasibility analyses (16). For 4Cs and field note analysis, each technique used was coded. Codes were then organized into a matrix of categories of therapeutic techniques that were based on a review of the literature and reviewed by a panel of experts in child mental health and education (Table 3). In line with a content analysis method, codes were tallied and tallies of codes within each category were summed to provide a descriptive picture of the types of techniques teachers chose for therapy delivery (42). ATLAS.ti was used for interview analysis and NVivo was used for 4Cs and field note analysis (43, 44).

**Table 3:** Therapeutic techniques categories and codes.

	**Categories of therapeutic techniques**					
	**Academic accommodations**	**Individual classroom behavior**		**Relationship-building**	**Self-regulation**	**Cognitive restructuring**
**Therapeutic Technique Codes**	Avoid timed tests	Avoid giving negative attention	Play as a reward	Check-ins	Baseline of feelings	Leadership
	Embed choices	Acknowledge only positive behavior	Giving specific rewards	Collaborate with family	Calming box	Challenging core beliefs
	Extra time	Alternate lunch	Praise	Interesting topics	Ask for a break	Power cards
	Less work	Avoid prolonged discussions	Praise for behavior	Lighten the moment	Coloring	Reframe thoughts
	Multisensory	Visual checklist	Praise for incremental steps	Narrate time with student	Emotion color chart	Self-esteem
	Present only a few items at a time	Give warnings	Miss breaktime	Non-contingent reinforcement	Emotion recognition	Balancing thoughts
	Preview work	Label the disagreement	Extra work	One on one tasks	Emotion thermometer	One on one when calm
	Reduce expected completed work	Non-preferred activity before a break	Praise other students	Polite language	Imagery	
	Reduce, accommodate, eliminate homework	Positive verbal reinforcement	Distance self from student	Positive attention	Music	
	Schedule preventative breaks	Praise incremental change	Contract	Validate feelings	Physical activity	
	Small group settings	Reinforce appropriate behavior	Simple reward system, unspecified	Games	Physical coping skills	
	Visual schedules	Simple language	Buddy system at recess	Consistent attention	Running to calm	
	Visual timer	Punishment	Sit with specific peers	Focus on interests	Self-regulation	
	Write out routine	Breaks outside the classroom	Praise in front of friends	Connecting through play	Stress ball to calm	
	Change in lesson	Change seating in classroom, unspecified location	Foster school climate	Eat lunch together	Ask for help	
	Leveling	Sit closer to teacher	5-minute reward	Asking about problems	Counting	
	Control and choice	Reward chart	Empowering statements	Guidance	Dancing	
	One on one education help	Read the room	Gentle, specific language	Help child save face	Breathing exercises	
	Focus on strengths			General conversation		
	Praise for academics					
	Reward for work					

## Results

### Demographics

Of participating teachers (*n* = 19), the majority were female (74%) and 27.76 years old on average (range 21–39) (Table 4). Forty-two students were nominated and consented to the intervention; five students were withdrawn pre-intervention as their teachers determined that their behaviors normalized and they no longer required additional support (Table 5). The remaining 37 children received care from their teachers, and the 36 children who completed all data collection were included in data analyses. The average age of enrolled children was 8.9 years and 41.7% were female. Their diagnostic categories of struggles ranged across all aggregate and subdomains measured by the TRF, indicating a wide variety of symptoms experienced by enrolled children (Table 6). The most frequently experienced symptoms were internalizing in nature (*n* = 19). Correspondingly, the most frequent categories of symptoms children experienced were in depressive subdomains; 11 children were classified as anxious/depressed and 16 children as withdrawn/depressed on syndrome scale scores while 14 children were classified as having depressive problems on DSM-oriented scales.

**Table 4:** Demographic Characteristics of Participating Teachers (*n* = 23)^a^.

**Characteristics**	
Age y, mean (range)	27.76 (21–39)
Years teaching at current school, mean (range)	4.2 (1–17)
Years teaching total, mean (range)	4.7 (1–17)
Female sex, No. (%)	17 (74)
Scheduled caste/scheduled tribe, No. (%)^b^	7 (30)
Grades taught, No. (%)^c^	
Class I (1st Grade)	22 (96)
Class II (2nd Grade)	2 (9)
Class III (3rd Grade)	21 (91)
Class IV (4th Grade)	16 (70)
Language, No. (%)^c^	
Nepali	22 (96)
Bengali	2 (9)
English	21 (91)
Hindi	16 (70)
Level of education, No. (%)	
Some primary	1 (4)
Some secondary	2 (9)
Finished secondary	2 (9)
Undergraduate or higher	18 (78)
Has formal training in education, No. (%)	4 (17)
Has certification in teaching No. (%)	3 (13)
Additional school responsibilities, No. (%)^c^	
Yes	6 (26)
Sports	3 (13)
Cultural	1 (4)
Accounting	1 (4)
Typing	1 (4)
Other employment, No. (%)^c^	
Yes	15 (65)
Housework	11 (48)
Selling things/running a shop	1 (4)
Farming/agriculture	4 (17)
NGO	1 (4)
Tour guide	1 (4)
Tutoring	1 (4)

*^a^Demographics for all 23 teachers who consented for the intervention are provided here. 19 teachers complete all study activities.*

*^b^Scheduled Caste and Scheduled Tribe are standard terms used in Indian demographic surveys referring to officially recognized groups of historically disadvantaged peoples by the Government of India and State of West Bengal.*

*^c^Sum is greater than 100% due to individuals teaching multiple grade levels, speaking multiple languages, having multiple additional school responsibilities, or having multiple forms of other employment*.

**Table 5:** Demographic characteristics of participating children (*n* = 36)^a^.

**Characteristics**	
Age y, mean (range)	8.9 (6–13)
Female sex, No. (%)	15 (41.7)
Scheduled caste/scheduled tribe, No. (%)^b^	11 (30.6)
Grade, No. (%)	
Class I (1st Grade)	10 (27.8)
Class II (2nd Grade)	8 (22.2)
Class III (3rd Grade)	8 (22.2)
Class IV (4th Grade)	10 (27.8)
Mother's education, No. (%)^c^	
Some primary	8 (22.9)
Primary	3 (8.6)
Secondary	23 (65.7)
Higher secondary	1 (2.9)
Undergraduate or higher	0 (0)
Father's education, No. (%)^d^	
Some primary	3 (9.1)
Primary	2 (6.1)
Secondary	25 (75.8)
Higher secondary	3 (9.1)
Undergraduate or higher	0 (0)
Monthly income (USD)^c, e^, mean (range)	151 (19–769)
Monthly income category (USD)^c, e^	
0–99 (%)	18 (51.4)
100–199 (%)	9 (25.7)
200–299 (%)	3 (8.6)
>299 (%)	5 (14.3)
Household size, mean (range)	4.4 (2–7)

*^a^Thirty-seven children received care. Data is missing for one child, allowing analyses of data from 36 children.*

*^b^Scheduled Caste and Scheduled Tribe are standard terms used in Indian demographic surveys for officially recognized groups of historically disadvantaged peoples by the Government of India and State of West Bengal.*

*^c^Data of 35 enrolled children were available and presented here.*

*^d^Data of 33 enrolled children were available and presented here.*

*^e^Monthly income as reported by family in 2019. Presented in USD (1 USD = 65 INR)*.

**Table 6:** Student mental health profile per the ASEBA TRF.

	**Symptom category**	Number of children with positive symptom score*^∧^
Aggregate scale scores	Internalizing problems^a^	19
	Externalizing problems^a^	11
	Sluggish cognitive tempo^b^	10
	Obsessive compulsive^b^	11
	Stress related problems^b^	17
Syndrome scale scores	Anxious/depressed^b^	11
	Somatic complaints^b^	7
	Thought problems^b^	6
	Attention problems^b^	6
	Rule-breaking behavior^b^	6
	Aggressive behavior^b^	8
	Withdrawn/ depressed^b^	16
	Social problems^b^	11
DSM oriented scales	Depressive problems^b^	14
	Anxiety problems^b^	9
	Somatic problems^b^	6
	Attention deficit^b^	6
	Oppositional defiant problems^b^	6
	Conduct problems^b^	8

*a positive score was defined as a borderline or clinical score as per TRF author guidelines.

*^∧^Children receiving care could have positive scores in more than 1 symptom category; N = 36 children completed all data collection and received care.*

*^a^Scores under the 83rd percentile were considered normal for the Total Problem, Internalizing and Externalizing aggregate scale scores.*

*^b^Scores under the 93rd percentile were considered normal for the remaining aggregate scale, syndrome scale, and DSM oriented scale scores*.

### Fidelity Outcomes

Participation rates for the assessed activities of behavior analysis and behavior plan were 85.7% and 81.0%, respectively. In time logs, teachers averaged 2.5 interactions/week and invested a mean of 19.0 min/week (standard deviation 27.5). The mean quality rating for each intervention activity met or exceeded the predefined benchmark: 60% for behavior analysis and behavior plan and 3 out of 5 for 1:1 student interaction and 1:1 family interaction (Table 7). Similarly, for individual observations, the percentage of teachers meeting or exceeding the benchmark ranged from 72 to 100%.

**Table 7:** Adherence and quality of delivery by intervention component.

**Intervention component**	**Quality rating (mean, SD)**	**Percentage of observations meeting or exceeding pre-defined benchmark (%, proportion)**
Behavior analysis^a^	0.68 (0.32)	72.22 (13/18)
Behavior plan^a^	0.87 (0.07)	100 (17/17)
1:1 student interaction^b^	3.00 (0.64)	79.17 (19/24)
1:1 family interaction^b^	3.29 (0.5)	100 (17/17)

*SD, standard deviation;1:1, one-on-one.*

*^a^Exceeding the predefined quality rating benchmark on these intervention components, 60% or 0.6, meant that the intervention component was delivered as intended and with high quality.*

*^b^Exceeding the predefined quality rating benchmark on these intervention components, 3 out of 5, meant that the intervention component was delivered as intended and with high quality*.

**Figure 2:**
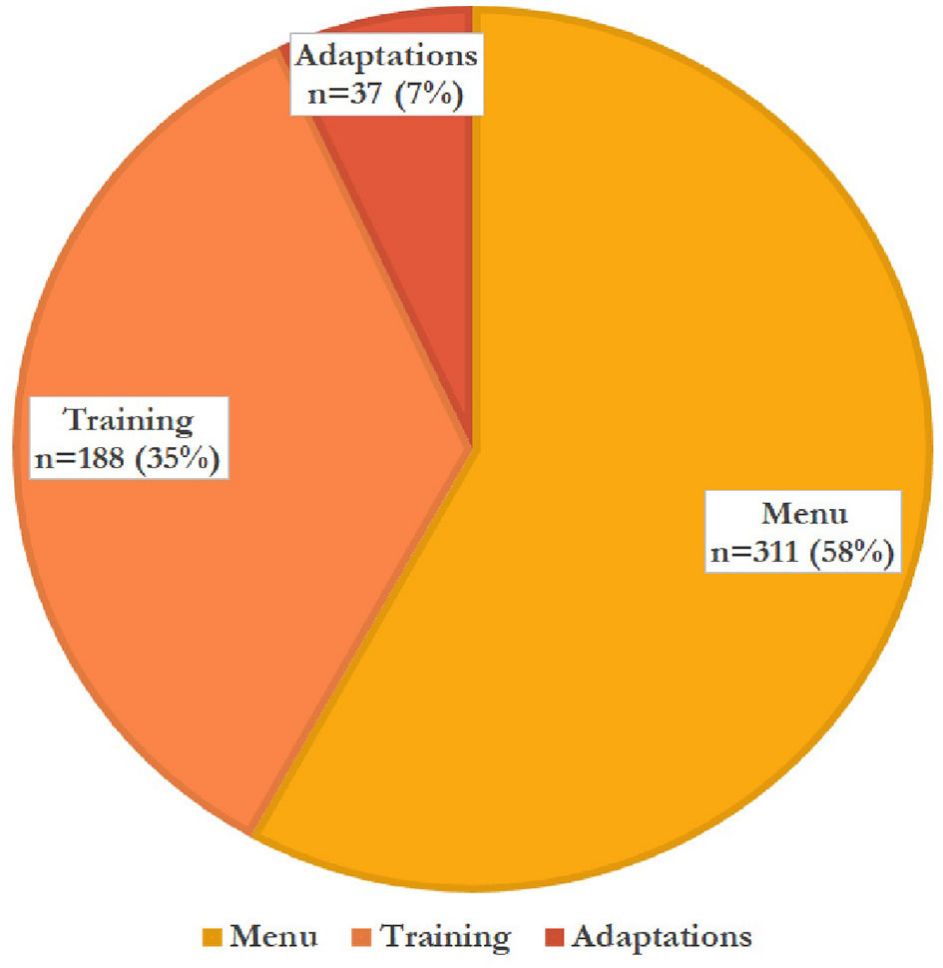
Menu, training, and adapted techniques used by teachers *n* = 536.

**Figure 3:**
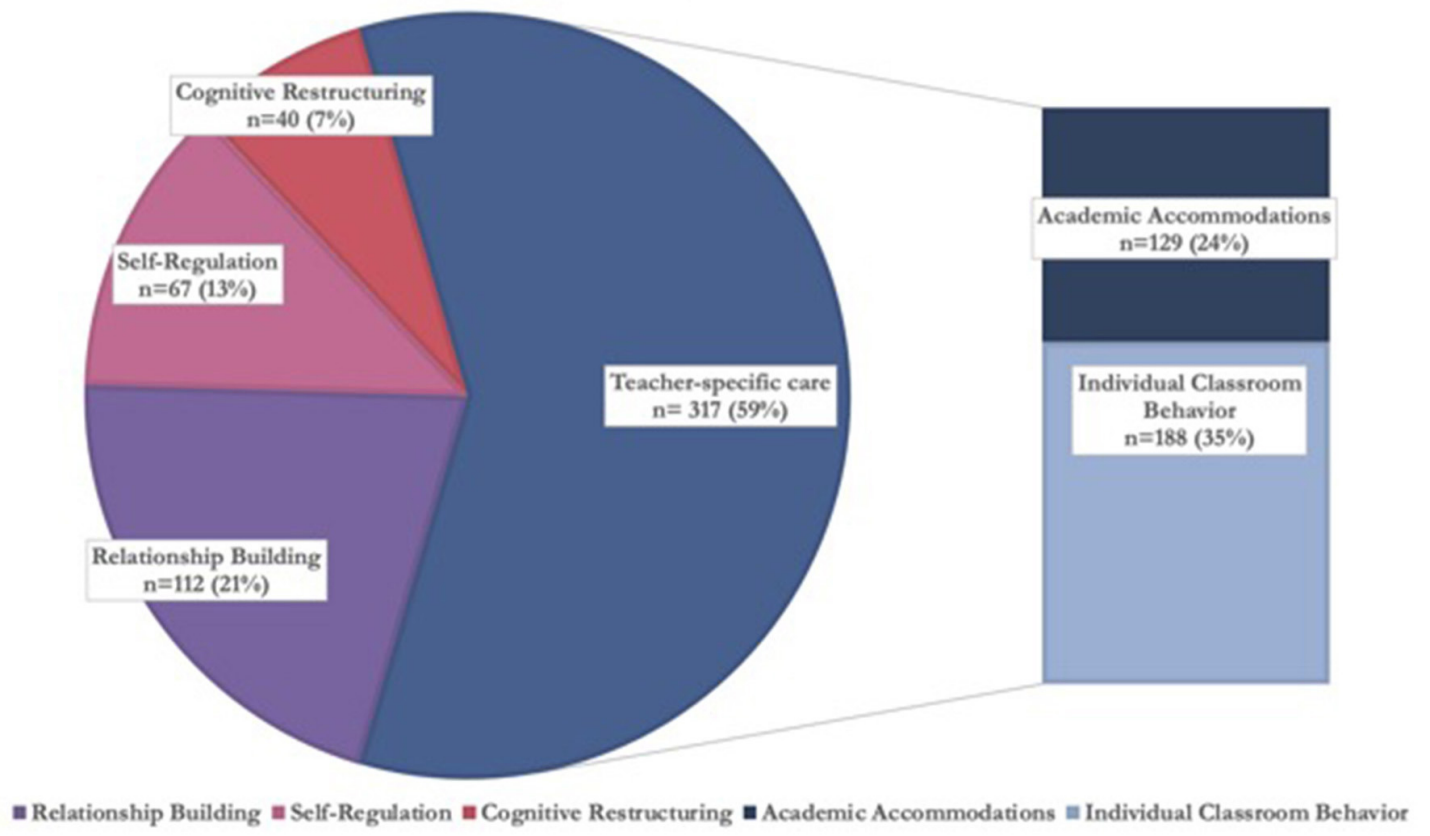
Categories of therapeutic techniques teachers used, grouped by teacher-specific care *n* = 536.

**Figure 4:**
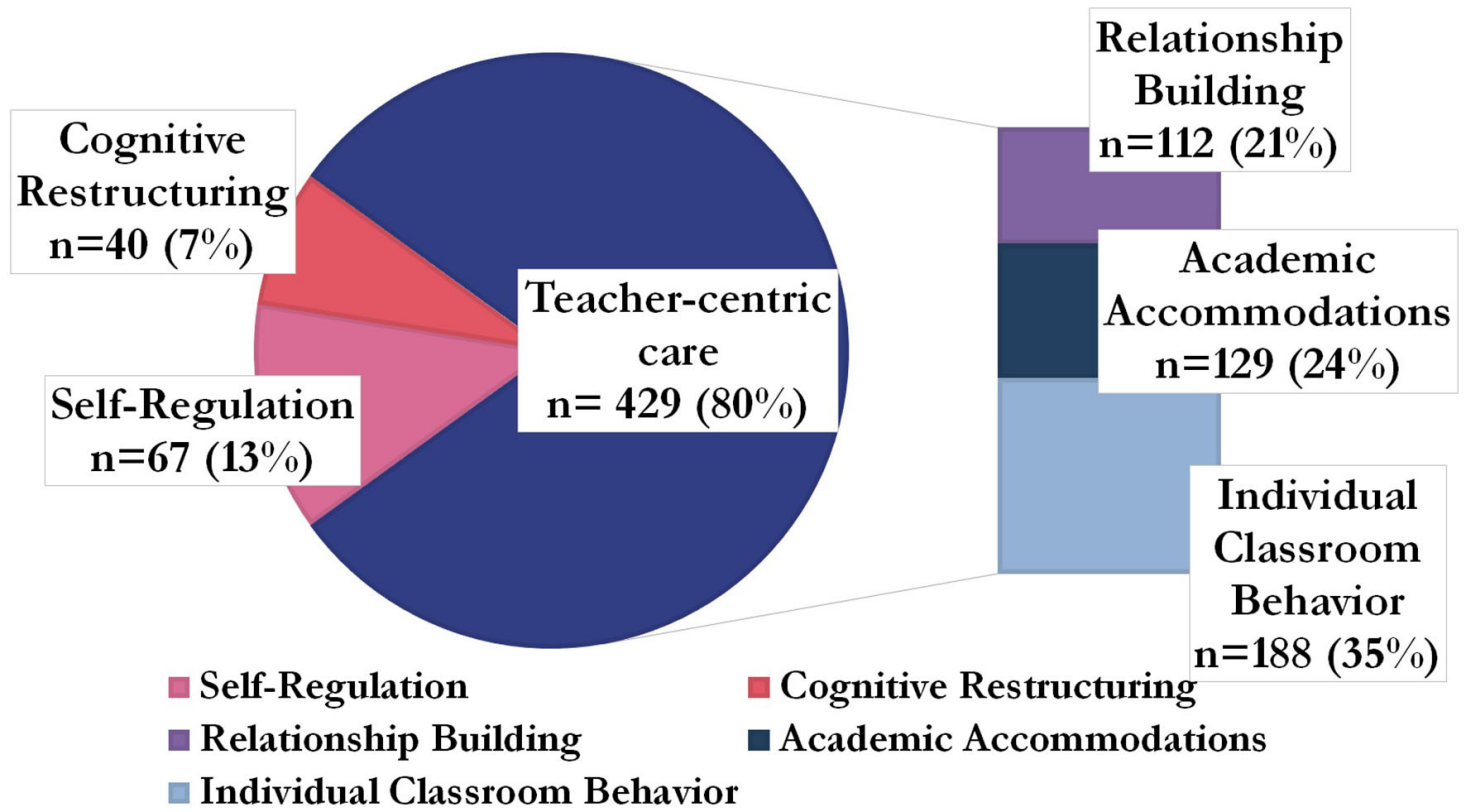
Categories of therapeutic techniques teachers used, grouped by teacher-centric care *n* = 536.

Figure 2 details the 536 therapeutic techniques teachers used per Tealeaf source (menu, training, or adaptation). Figures 3, 4 detail the 536 techniques per category (academic accommodations, individual classroom behavior, relationship building, self-regulation, or cognitive restructuring). Three-hundred-seventeen techniques (59%) were those only teachers (not office-based therapists) would be able to deliver, here termed “teacher-specific” care (Figure 3). These techniques had the purposes of improving both mental health and either knowledge transfer (“academic accommodations”) or classroom behavior (“individual classroom management”), tasks only teachers could dually accomplish and that office-based therapists could not, based on their job duties (8).

Academic accommodations (*n* = 129, 24%) were one category of techniques only teachers could use as only teachers would be able to use a student's academic work as a means of improving the student's mental health. A specific example of an academic accommodation technique follows. A student with anxiety who does not complete homework assignments is found to have counterproductive automatic thoughts underlying his homework difficulties, where he thinks he is incapable of completing homework assignments due to being overwhelmed by the quantity and needing to get all answers correct. To counter these automatic thoughts, his teacher can reduce the student's homework quantity for the purposes of behavioral activation, allowing the student to complete assignments successfully and counter the automatic thought that he is incapable. Over time, the teacher can increase the homework quantity as a form of exposure until the student can complete all of the originally assigned work. The second category of teacher-specific techniques centered on individual classroom behavior management (*n* = 188, 35%). For example, a teacher can separate the classroom and lunchroom seating of a student with depression and feelings of worthlessness from a student who is bullying the student and contributing to feelings of worthlessness, thereby reducing a precipitating factor of the student's experiencing of depression and making it easier for the student to practice self-regulation.

The relationship building techniques teachers used (*n* = 112, 21%) also consisted of measures only teachers would be able to take, such as giving consistent, non-contingent attention to the student receiving care throughout the school day. This was not counted in teacher-specific care, however, as office-based therapists also build relationships with clients as part of typical duties, though use different techniques (45). Still, when assessing the number of techniques teachers used that were ones only they could use, whether the purpose was unique to teachers (i.e., knowledge transfer or classroom behavior) or not (i.e., relationship building), here termed “teacher-centric,” 429 techniques (80%) used could be characterized as “teacher-centric” (Figure 4).

### Feasibility Findings

Interviews provide evidence that teachers find delivering mental health care as structured in Tealeaf and using Tealeaf program tools to be feasible (Table 8). Feasibility was facilitated by training and supervision, teacher ability to adapt Tealeaf, caregiver trust and engagement, and a belief that the program was impactful. Barriers to feasibility included a lack of time to deliver Tealeaf, difficulty translating the training to the classroom, and a lack of family engagement. A theme of acceptability was indirectly expressed by many teachers (Table 8). They discussed their continuous delivery of care throughout the study, a desire to serve in the program and deliver care after the study's conclusion, and advocacy for the program to expand beyond this study, signs of acceptability (46).

**Table 8:** Feasibility themes and sub-themes.

**Feasibility aspects**	**Theme**	**Sub-themes**	**Quotation**
Feasibility	Implementing mental health care delivery	Identifying children for mental health support	“*I had thought of one child and when the time came to identify one who needed help, I ended up choosing that child. In the training, we had learned to identify the qualities of a withdrawn child. This child possessed these qualities. So, I knew my choice was correct.” **-*****Teacher 75**
		Finding a root cause	“*Earlier, when the child would come to class without doing the homework, we would punish and scold them and label them as the ones who never do it and partially blame it on the parents. We never looked deeper than that. However, after the training we realized that we need to observe and find out the root cause for the behavior and the action of the child. After we started observing and asking around, we got to know that the child couldn't do the homework or study at home because there was no electricity at home. This way we became aware.”* **-Teacher 26**
		Building rapport with students	“*The first thing that I want to make sure is that my child isn't feeling nervous. So, I divert their mind by talking about things, and I look at their faces to see if they are relaxed or still tensed. I feel it is important for my child to feel relaxed and not scared or nervous.”* **-Teacher 80**
		Using therapeutic techniques	“*I used tools to calm them down and also referred to the physical coping strategies such as taking long deep breaths to help them calm down and cope with the situation.”* -**Teacher 99**
	Implementing program tools	AABC Chart	“*It didn't take me much time because even if I hadn't carried the chart with me and I happened to make an observation of the child at that moment, I would go back home or to my room and fill in the chart later on.”*-**Teacher 31** “*I found the chart to be good. The chart helped us notice or observe certain things about the child. And the chart helps us note down and summarize certain behavior about the child too.” -***Teacher 15** “*The ABC chart was okay. It has a limit setting section- I found that to be challenging.” **-*****Teacher 99**
		Relationship Building	“*I felt 4Cs to be important, and it was very useful and worked well too in my case. It was helpful in building connections with the children. I realized that you cannot build connection with the child by creating fear in their minds. There are other better ways to build and create the connection with the child that will make the child open up to you.”*-**Teacher 98**
		4Cs	“*Through the plan, we could bring about some improvement in the child. Some of the inadequate behavior was changed for the better. We cannot bring about 100% change in their behavior, but I know we have brought about 80% change in them.”* **-Teacher 23** “*Used things from the 4Cs plan and also did the one-on-one interaction with them. I used tools to calm them down and also referred to the physical coping strategies such as taking long deep breaths to help them calm down and cope with the situation. I also used the stickers and other charts and gave them prizes and praised them and even made them the monitor of the school.”* **-Teacher 27**
Feasibility facilitators	Training and Supervision		“*It is because of the training that we understood how to recognize and to handle such kinds of children.”* **-Teacher 27** “*I found it good. [The supervising psychiatric social worker] would come and tell us to do this and that because we wouldn't have the knowledge on how to go about certain things. So, I found it to be good.”*-**Teacher 22**
	Teacher adaptations		“*What I do is that I make sure the child does not realize the one-on-one session has started. I usually tell the child to get me something or help me with some work toward the end of the period so that the work would continue toward the recess period. This way the other children of the class would not suspect anything, and the child concerned also won't know that the one-on-one session has started.”* **-Teacher 80**
	Caregiver trust and engagement in program		“*Whenever I would meet them and talk, or even during a phone call, the parents would honestly tell me about their child and have a nice talk with me.”*-**Teacher 96**
	Belief program was impactful	Academics	“*As you saw, today was the report day, and the child got 2nd division and even received a prize. Seeing this makes me happy.”* **-Teacher 23**
		Teacher beliefs about mental health	“*Earlier I would not click with the students, I would go teach and come out, but now I mix with the students and get along with them. After the training I realized that our job is not confined to teaching, it is more than that. We need to realize that children too have problems, like [Student 22]. Earlier, I would punish or beat her when she would get talkative, but I realized that was not the way to handle it.”*-**Teacher 22** “*Earlier we would very easily punish the child without trying to find out the reason for his/her actions. But now we know that there are issues that lead a child to do that mischief or there is a reason why the child is behaving in that manner. We are much more aware and know better than to punish, scold or beat the child up.”*-**Teacher 31**
		Behaviors	“*The children started to talk and open up to the teachers, and when asked if there was something they hadn't understood, the children started to speak up and make conversations.” -***Teacher 95**
Feasibility barriers	Lack of time		“*I had gotten extremely busy by the time midterm started, so hardly any time could be given to this work, and then it got really tedious for me and I couldn't give much attention and focus to what I was doing with the chart and the child.” -***Teacher 26**
	Difficulty translating training to the classroom		“*Initially when we did the training, we found it to be really easy, but after the training when we had to fill the chart of the students at the end of the session, then we found it challenging, and it was way more difficult than the training that we received.”* *-***Teacher 26** “*Interviewer: In what areas did you want the extra training?* *Teacher: Things that will help tell us why is the student not studying well or why is the child failing to do the given homework and all of that.”* **-Teacher 96**
	Lack of family engagement, communication, or understanding		“*It is difficult to coordinate and take time out of the day to meet the parents. These parents are in the villages so it is far for them to come at a certain time and most of the time they have a lot of things on their plate so they can't make it to the meetings.”* **-Teacher 85**
Acceptability	Program		“*I still implement [the program tools] to date and it gives really good results.”*-**Teacher 57** “*Overall, it's been a good experience with the children.”*-**Teacher 13**

### Exploration of Impact; Correlation Between Fidelity and Impact

Improvement in mental health status is demonstrated by the decrease in mean TRF Total Problem score percentile across three time points (Table 9 and Supplementary Figure 8). Primary observers rated children on average as increasing in normalcy and secondary observers rated children on average as improving from borderline to normal. Only total time a teacher spent on intervention and their student's mental health outcomes were correlated (Pearson Correlation Coefficient 0.46, *P* = 0.009; Supplementary Table 1).

**Table 9:** Children's mental health outcomes.

**Observer**	**Baseline, mean (SE)**	**Midpoint, mean (SE)**	**Endpoint, mean (SE)**	**β^a^**	P-value*
Primary observer	77.51 (3.47)	68.49 (4.22)	60.09 (4.98)	−8.65	0.005*
Secondary observer	87.23 (3.31)	62.17 (3.84)	61.46 (4.38)	−12.86	<0.001*

*Mean scores on the TRF total problem scale are expressed as percentile scores. Standard scores are normed to the 50^th^ percentile. Thus, for scores above the 50^th^ percentile, a decrease in scores indicates less deviance from normality (i.e. improved mental wellbeing). The primary observer was the teacher providing intervention support and the secondary observer was an independent teacher familiar with the child's behaviors.*

*^a^β represents the slope for the linear regression comparing endpoint to baseline scores, adjusted for gender.*

*SE, standard error.*

*p < 0.05 is considered significant.

## Discussion

Overall, the results of this mixed-methods feasibility study suggest that teachers in resource-limited primary schools in an LMIC setting are able to deliver indicated, transdiagnostic child mental health care with fidelity and feasibly but in a particular form, one with flexibility and that is incorporated into a teacher's primary duty of teaching.

Implementation fidelity underlies whether and how interventions achieve their intended outcomes, but may be at odds with feasibility (47). Teachers achieved quality implementation and were witnessed to adopt their chosen therapeutic techniques into their interactions with students and caregivers. Some teachers expressed difficulty with specific intervention tools. Overall, though, in interviews most teachers did not perceive Tealeaf to be beyond their capacity or abilities, and many further expressed acceptability of Tealeaf, indicators that teachers viewed delivering care with fidelity to be feasible.

The feasibility of delivering Tealeaf with fidelity may have been enhanced by teachers' ability to choose and adapt which therapeutic techniques to use. Fidelity is viewed as a marker of high quality implementation and is often in opposition to choice and adaptation (48). Here, with evidence of teacher choice considered a positive marker of fidelity, a balance of fidelity with choice and adaptation was intentionally sought. In unpublished pilot testing results, teachers expressed both that Tealeaf techniques were usable and that they valued the ability to choose and adapt techniques as seen fit. In this study, teachers then demonstrated that delivering Tealeaf care with fidelity was considered feasible while also using a wide variety of techniques. Further, in interviews, they voiced the usability of Tealeaf tools while also expressing that choice and adaptation were crucial to facilitating feasibility. Thus, it may be that a balance between fidelity to a structure (such as use of the 4Cs behavior plan) and choice and adaptation is needed to allow teachers to feasibly deliver care on top of and that fits with their typical duties.

The novel finding here is that, as a group, teachers organically overwhelmingly chose “teacher-centric” techniques (80%) to deliver child mental health care, that is, techniques that only teachers, and not office-based therapists, could implement. By contrast, we hypothesized that teacher-centric techniques would be a supplement to, but not more predominant than, one-on-one sessions. Such a consistent choice makes the care delivered in this study predominantly classroom-based and rooted in the knowledge transfer process, a sharp divergence from traditional office-like one-on-one sessions. An earlier publication from this group of authors found evidence for the potential emergence of “education as mental health therapy,” here abbreviated as “Ed-MH,” where, in interviews, teachers consistently reported choosing to use the mental health techniques that they could incorporate into the knowledge transfer process, their primary duty (32). In the present study, we believe we find evidence corroborating the emergence of Ed-MH as a potentially new therapy modality: teachers consistently chose to deliver care that was teacher-specific 59% of the time and teacher-centric 80% of the time, care only they could deliver.

The organic emergence of Ed-MH may speak to task-shifted care delivery by teachers having to be incorporated into their primary duties to be feasible. Time log results further support this consideration. The time logged in one-on-one sessions may not have included the total time teachers spent delivering care, as the time log did not account for the time teachers spent using teacher-centric techniques (Supplementary Figure 5) (6). Instead, the time logged may be the average available weekly time teachers could spend in one-on-one sessions, below our expectations. Further, a theme of a lack of time emerged in teacher interviews as a barrier to feasibility for some. Thus, for teachers to feasibly deliver indicated care on top of their regular duties, it may need to be incorporated into their workflow.

It is possible that teachers used one-on-one sessions less frequently than teacher-centric techniques not because of the additional time needed to conduct these sessions, but instead because they were less familiar with them. Accordingly, teachers may have chosen to use teacher-centric techniques for their familiarity. Moreover, in interviews, some teachers mentioned wanting more time in training while others stated wanting more frequent supervision (as under the theme “Difficulty translating training to the classroom” in Table 6). However, most teachers sought additional guidance on use of teacher-centric techniques that were nevertheless novel to them despite their overlap with methods of teaching. In the setting of time being a barrier to feasibility for some, then, it may be that teachers have additional time and capacity to learn therapeutic techniques that are closely related to their primary duties as these techniques can be relatively efficiently learned. By contrast, the knowledge and skills teachers need to conduct one-on-one sessions are conceptually further afield from their primary duties, potentially requiring further additional time to learn them that teachers do not have to spare. Teachers delivering care primarily through one-on-one sessions, thus, may be less feasible than an Ed-MH model given the additional time needed both to conduct the sessions and to learn the skills needed for these sessions, even if the latter are potentially attainable through additional training and supervision.

Notably, even when likely underestimated, time was the only fidelity factor correlated with improved child mental health outcomes. It may be that the extended time teachers can spend with children with mental health needs throughout the school day could be among the more effective structures through which to deliver task-shifted care. The literature correspondingly points to increased academic learning time in classrooms being associated with improved learning; a dose-response relationship between child psychotherapy sessions and mental health outcomes, though, has not been evidenced in the literature, perhaps speaking to the mixed efficacy of task-shifted child mental health care that has predominantly been structured as one-on-one sessions (49–51).

The teacher-centric techniques of Ed-MH, akin to an educational intervention, overlap with adaptations to pedagogy teachers make to individually target different learners, a standard in education (52). As above, teacher-centric techniques may have been predominantly chosen for their familiar overlap with pedagogy (52). Ed-MH differs from individualized pedagogy, however, in that its primary goal is to improve mental health, while individualized pedagogy solely aims to successfully transfer knowledge to the student (52). Evidence in the literature points to a potentially marked difference between Ed-MH and individualized pedagogy. Interventions enhancing teachers' ability to individualize pedagogy through additional training have demonstrated meaningful improvement in student academic, but not mental health, outcomes (53, 54). By contrast, in this study, Ed-MH showed a potential signal of efficacy with improved TRF Total Problem percentiles over the year.

This study expands on the current literature by demonstrating that teachers can deliver indicated mental health care to children in need when incorporating the care into their workflows as in Ed-MH within Tealeaf, in line with other studies reporting that teachers can deliver care with whole-school and whole-classroom-based aspects feasibly and with impact (11, 12). The Ed-MH structure is an alternative to the traditional and validated structure of task-shifted mental health care, modeled after office-based care (i.e., one-on-one sessions) in which lay counselors' main duty is to provide care. While such a model has been validated, it may not be sustainable as it requires employing additional human resources (4, 6, 11, 12, 46). Leveraging teachers to deliver care through interweaving it into their teaching duties, by contrast, may lead to the eventual sustainability of task-shifted child mental health care as it can feasibly and efficiently be delivered by an existing, experienced human resource within their existing workflows (28).

### Limitations

Our findings should be considered within a number of limitations. First, due to the novel nature of the intervention, we were unable to identify suitable pre-existing validated fidelity measures and instead used study-specific instruments. Second, observational assessments were conducted by the same individual who trained and supervised the teachers. Resource limitations prevented the use of external raters. To mitigate potential biases we (1) utilized several strategies to promote ratings reliability and (2) purposely set quality benchmarks to be high, both discussed previously. Third, findings from a separate publication indicate that teachers identified more students in need of mental health care than they were able to nominate based on pragmatically set limitations (21, 22) and, based on resource limitation, we were unable to examine whether teachers could feasibly deliver care to more than two children. A future effectiveness and/or implementation study is warranted to examine whether teachers can feasibly deliver care to more than two children and whether mental health symptoms improve to the same extent for these children whose teachers are delivering care to more than two children. Such a study would further elucidate the potential reach of teachers as lay counselors. Fourth, our time analysis is limited by the quality of data captured by the teachers, previously discussed. Fifth, the TRF may not have accurately captured child mental health statuses in the Darjeeling context, as described earlier. A publication from this group of authors showed that a majority of children from Darjeeling nominated by teachers for mental health support had “normal” TRF Total Problem Scores, close to the borderline/normal cutoff; the selected students instead had “borderline” or “clinical” subscale scores reflecting teachers' concerns for their mental health needs to the point of categorizing most of them as “definitely needs support” on the BTST (21). Still, the TRF was chosen for its relative strength in local applicability and validation as judged by a panel of experts (37–39). Sixth, positive mental health outcomes are preliminary. Obtained in a small sample size, they were not compared to controls and the time interval between measurements raises the possibility of regression to the mean (55). Further, they were completed by teachers, subject to bias in wanting their students to improve, though mitigated some by a second teacher not working with the child additionally rating the students' mental health.

Findings may not be generalizable to different regions or types of schools (such as government). Specific aspects of the teacher population, such as having limited prior education training, may have made them more receptive to training than more established educators and may limit our findings' generalizability (56). Additionally, teachers were not randomly chosen and may have been more enthusiastic to deliver care than teachers more broadly.

## Conclusion

Child mental health remains under-developed within the larger global mental health movement. The vast majority of children in LMICs lack access to high-quality mental health care. Findings from this study suggest that Ed-MH delivered within Tealeaf is potentially an efficient and sustainable path forward for bridging the care gap.

Continued innovation and research around teacher-delivered transdiagnostic mental health care may thus be warranted. Examining the feasibility and acceptability of teacher-delivered care to children and their families will crucially inform whether potential recipients of care are willing to receive it in a relatively public space (the classroom) from a community member of import culturally (57). Examining the acceptability to teachers of their delivery of care to their students merits evaluation as having a dual role may create conflict with their students and families and/or break trust between them. These concerns are the subject of a separate manuscript under peer review from this group of authors. Further, our group is conducting a cluster randomized controlled trial to definitively examine child mental health outcomes after receiving teacher-delivered Ed-MH mental health care in Tealeaf. Ultimately, such work has the potential to improve the life trajectories of children with a broad range of mental health challenges globally, wherever teachers teach.

## Data Availability Statement

The datasets generated and/or analyzed during the current study are not publicly available for ethical reasons. Requests to access the datasets should be directed to christina_cruz@med.unc.edu.

## Ethics Statement

The studies involving human participants were reviewed and approved by the University of North Carolina at Chapel Hill Institutional Review Board and a Darjeeling-based Ethics Committee. Written informed consent to participate in this study was provided by the participant or, for minors, their legal guardian/next of kin, as follows.

- Schools: PG called principals of area schools to gauge interest. Interested principals discussed with their teachers their interest in intervention delivery and study participation.
- Teachers: All eligible teachers in participating schools were invited to meet with study representatives to review study protocols. Those interested in participating voluntarily signed a written informed consent.
- Children: Parents or guardians of children nominated by their teacher for the intervention were invited to meet with study representatives to review study protocols. Those interested in their children participating in the study voluntarily signed a written informed consent on behalf of their children. Children greater than 7 years of age were verbally assented for participating in the study and were allowed to refuse to participate.

## Author Contributions

CMC, KH, and MM designed the study. BNG was involved in study design. CMC, PG, and MM created the teacher training, intervention materials, and intervention protocol. PG delivered the teacher training, provided supervision to teachers, and collected data. SB provided supervision to teachers and collected data. CMC, AAG, and MM provided umbrella supervision for the supervision of teachers by PG and SB. MML performed quantitative data analysis while JLV and PF performed qualitative data analysis. CMC, MML, KH, PG, SB, JLV, PF, AAG, and MM were involved in data interpretation. CMC, PG, MML, JLV, and MM drafted the manuscript. All authors revised and approved the final version of the manuscript before submission.

## Funding

This work was generously supported by the American Association of Child and Adolescent Psychiatry (AACAP) through the Pilot Research Award for Junior Faculty and Child and Adolescent Psychiatry Fellows, supported by AACAP, awarded to CMC; the manuscript's contents are the responsibility of the authors and do not necessarily reflect the official views of AACAP. The publication of findings is made possible by the Doris Duke Charitable Trust, Fund to Retain Clinical Scientists—Caregivers at Carolina Award, awarded to CMC.

## Conflict of Interest

CMC, PG, and MM hold the copyright to the training materials, decision support tools, and intervention materials for the teacher-led task-shifted alternative system of children's mental health care at the center of this manuscript. They have disclosed this interest fully to Frontiers in Psychiatry. The remaining authors declare that the research was conducted in the absence of any commercial or financial relationships that could be construed as a potential conflict of interest.

## Publisher's Note

All claims expressed in this article are solely those of the authors and do not necessarily represent those of their affiliated organizations, or those of the publisher, the editors and the reviewers. Any product that may be evaluated in this article, or claim that may be made by its manufacturer, is not guaranteed or endorsed by the publisher.
