# Erratum: The Potential Emergence of “Education as Mental Health Therapy” as a Feasible Form of Teacher-Delivered Child Mental Health Care in a Low and Middle Income Country: A Mixed Methods Pragmatic Pilot Study

**DOI:** 10.3389/fpsyt.2021.838044

**Published:** 2022-01-18

**Authors:** Frontiers Production Office

**Affiliations:** Frontiers Media SA, Lausanne, Switzerland

**Keywords:** teacher, task-shifting, child mental health, feasibility, fidelity, global mental health, school mental health, education as mental health therapy

Due to a production error, there were mistakes in Figures 2 and 4 as published. Incomplete versions of the figures were used, which did not contain the full data. The corrected Figures 2 and 4 have been updated and appear below.

**Figure 2:**
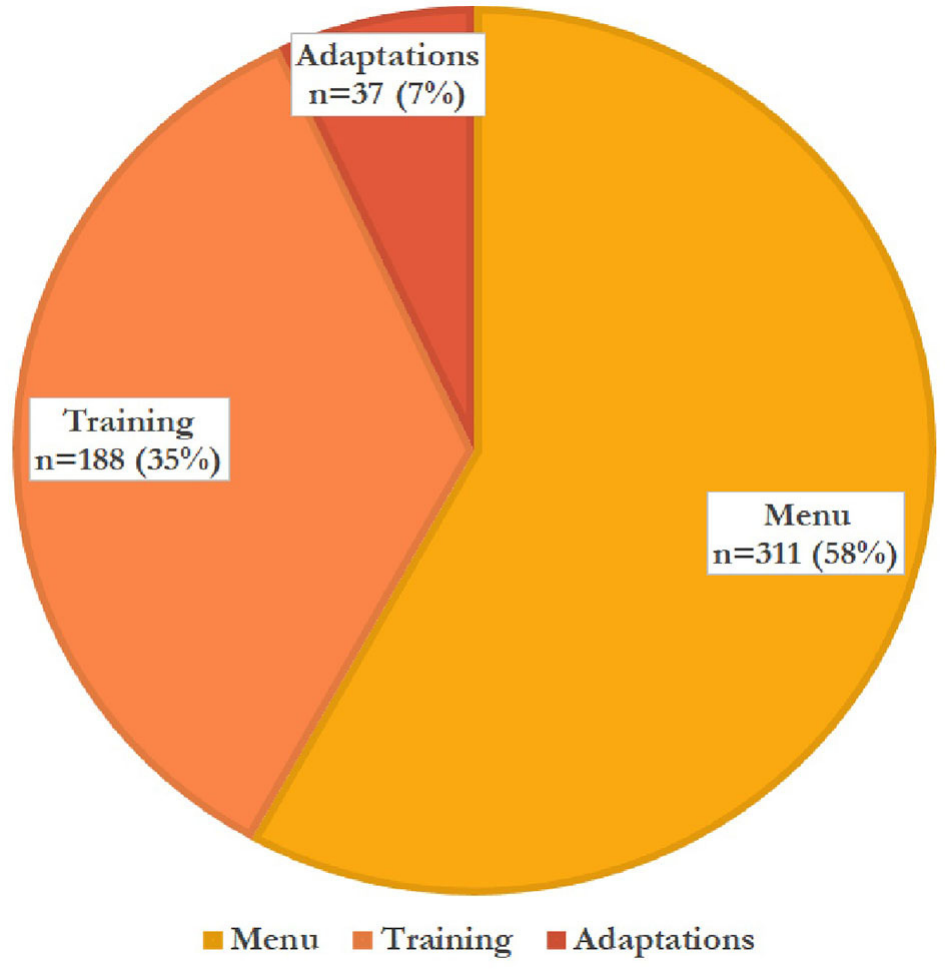
Menu, training, and adapted techniques used by teachers *n* = 536.

**Figure 4:**
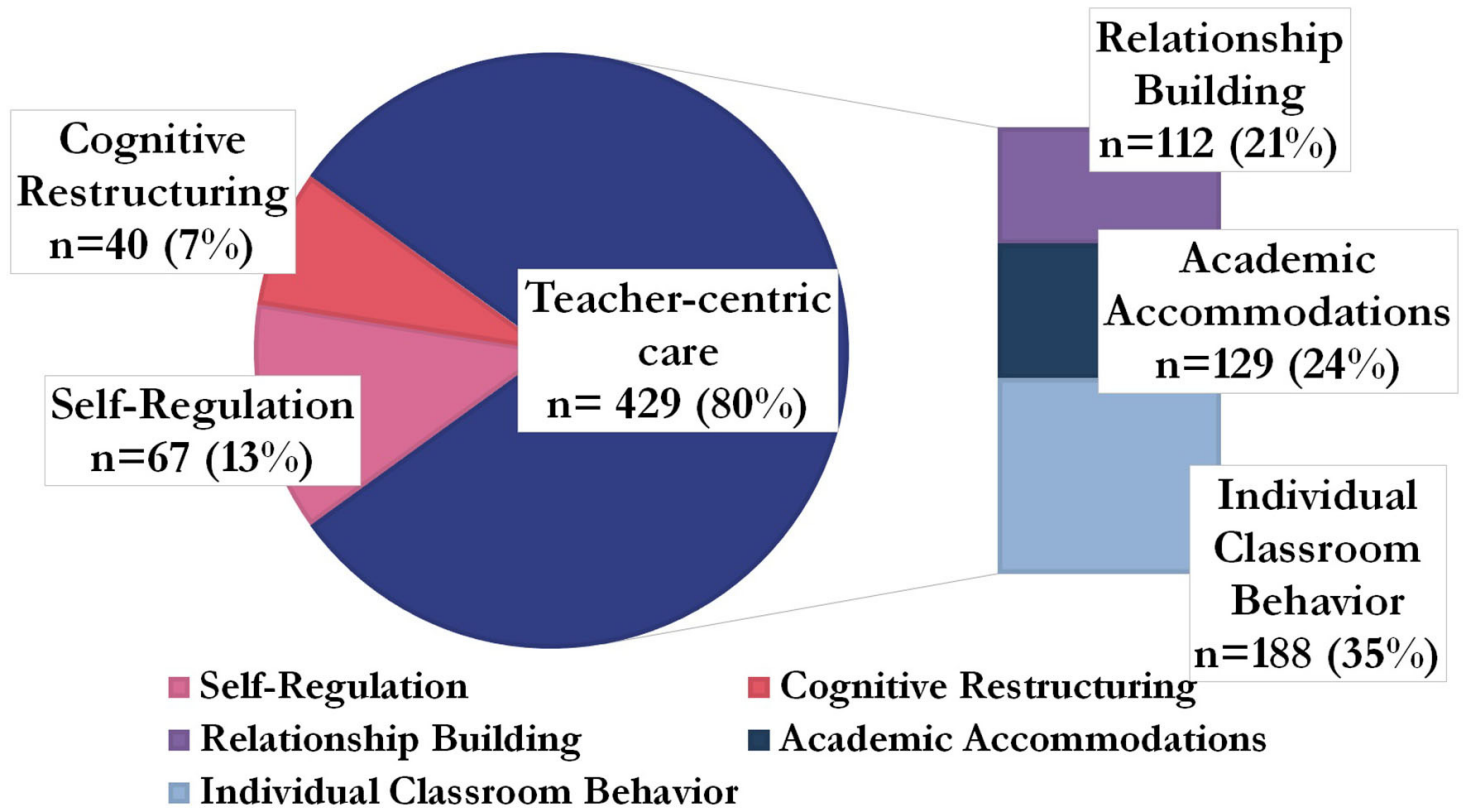
Categories of therapeutic techniques teachers used, grouped by teacher-centric care *n* = 536.

The publisher apologizes for these mistakes. The original article has been updated.

## Publisher's Note

All claims expressed in this article are solely those of the authors and do not necessarily represent those of their affiliated organizations, or those of the publisher, the editors and the reviewers. Any product that may be evaluated in this article, or claim that may be made by its manufacturer, is not guaranteed or endorsed by the publisher.

