# Supplementary material for: The Potential Emergence of “Education as Mental Health Therapy” as a Feasible Form of Teacher-Delivered Child Mental Health Care in a Low and Middle Income Country: A Mixed Methods Pragmatic Pilot Study
Source: Front Psychiatry. 2021 Dec 16;12:790536. doi: 10.3389/fpsyt.2021.790536 (PMC8717545; doi:10.3389/fpsyt.2021.790536)
Supplement: Supplementary file 1 [file Data_Sheet_1.docx]

**Supplementary Figure 1.** Behavior Type and Severity Scale (BTST)

Teacher Study ID: __________

Class (please circle): I (1) II (2) III (3) IV (4)

*Note: please use only one form per class level.*

Use the behavior rating scale below to classify the behavior of each of your students; record this number in the column labelled “Behavior Rating Scale Number”. Rate each student on a scale of 1 to 9 in terms of their behavior, where 1 is the healthiest behavior and 9 is the most impaired. Students you rank 1-3 are generally students who do not need extra support. Students you rank 4-6 might benefit from support but may also not need it to do well. Students you rank 7-9 definitely need support to do well.

Then, only if appropriate, state whether you believe the student has anxious, disagreeable, or withdrawn behavior in the column “Type of Behavior”. Only record this for students who exhibit these types of behaviors. Some students will not exhibit these types of behaviors and thus will not need for you to state this. If you feel the student has more than 1 type of behavior, rank the behaviors from 1-3, where 1 is the most common behavior for the student.

Recall that anxious behavior is based in an overwhelming and abnormal sense of apprehension, worry, or nervousness. Disagreeable behavior is rooted in needing a sense of control and/or having difficulty with being flexible. Withdrawn behavior comes from not being able to participate in one’s everyday activities.

1

2

3

4

5

6

7

8

9

Healthy

Impaired

**Behavior Rating Scale**

**Does not need support**

**Might need support**

**Definitely needs support**

| **Student Name** | **Behavior Rating Scale Number** | **Type of Behavior** |
| --- | --- | --- |
|  |  |  |
|  |  |  |
