# Supplementary material for: The Potential Emergence of “Education as Mental Health Therapy” as a Feasible Form of Teacher-Delivered Child Mental Health Care in a Low and Middle Income Country: A Mixed Methods Pragmatic Pilot Study
Source: Front Psychiatry. 2021 Dec 16;12:790536. doi: 10.3389/fpsyt.2021.790536 (PMC8717545; doi:10.3389/fpsyt.2021.790536)
Supplement: Supplementary file 4 [file Data_Sheet_4.docx]

**_Supplementary Figure 4_**_. Menus of Therapeutic Techniques, per Behavior Category_

|  | **_Nervous_** | **_Disagreeable_** | **_Withdrawn_** |
| --- | --- | --- | --- |
| **_Cause_** | - _AABC Chart_ - _Themes of the AABC Chart_   - _Attention_   - _Escape_   - _Tangible_   - _Sensory_ | - _AABC Chart_ - _Themes of the AABC Chart_   - _Attention_   - _Escape_   - _Tangible_   - _Sensory_ | - _AABC Chart_ - _Themes of the AABC Chart_   - _Attention_   - _Escape_   - _Tangible_   - _Sensory_ |
| **_Change_** | - _School environment_   - _Schedule preventative breaks_   - _Small group settings_ - _Organization/planning_   - _Read the room_   - _Visual timers_   - _Avoid timed tests and games_   - _Visual schedules_   - _Schedule time to organize belongings_ - _Classroom learning_   - _Pre-determined visual cues for writing_   - _Use computers or mobile phones for word processing when subject is not penmanship_   - _Spelling accommodations when subject is not spelling_   - _Checklist of weakness and strategies to use in the moment_   - _Reduce expected completed work_   - _Preview work for the day for 5 minutes before the day starts_   - _Reduce, accommodate, or eliminate homework_ - _New skills_   - _Emotion recognition_     - _1:1 when calm_     - _In the moment_     - _Emotion color chart_     - _Emotion thermometer_   - _Linking emotion to behavior_     - _CBPT or CBT_   - _Self – regulation_     - _Calming box_     - _Physical coping strategies_     - _Imagery_     - _Music_     - _Coloring_   - _Targeting automatic thoughts and feelings_     - _Thought stopping (CBT)_     - _Power cards_   - _Challenging core beliefs_   - _Self-monitoring_ | - _School environment_   - _Schedule preventative breaks_   - _Daily schedule which alternates preferred and non-preferred activities_   - _Alternate lunch and/or recess_   - _Purpose during transitions_   - _Give warnings for end of activity and then start of next activity_   - _Distraction during downtime_ - _Organization/planning_   - _Visual timers_   - _Visual schedules and preview them_ - _Classroom learning_   - _Embed choice into the learning_   - _Reduce, accommodate, or eliminate homework_ - _New skills_   - _Emotion recognition_   - _Self – regulation_     - _Calming box_     - _Physical coping strategies_     - _Imagery_     - _Music_     - _Coloring_   - _Targeting automatic thoughts and feelings_     - _Power cards_   - _Challenging core beliefs_     - _Being Responsible worksheet_ | - _School environment_   - _Calming box_   - _Buddy system at recess_   - _Recess plan_   - _Alternate lunch and/or recess_ - _Organization/planning_   - _Checklist_   - _Visual schedules_   - _Visual timer_   - _Present only a few problems at a time_ - _Classroom learning_   - _Make lesson multi-sensory_   - _Classroom assignments on topics interesting to the withdrawn student_   - _Reduce, accommodate, or eliminate homework_   - _Extra time to complete exams and extra day(s) to complete homework assignments._ - _New skills_   - _Emotion recognition_     - _Color chart with added self-regulation checklist_     - _Daily self-reflection sheet_   - _Self – regulation_     - _Calming box_     - _Physical coping strategies_     - _Imagery_     - _Music_     - _Coloring_     - _Desk skills_     - _Heavy physical activity_   - _Reframe negative thoughts_     - _Thought stopping_     - _Balancing negative thoughts/feelings with positive ones_     - _Power cards_   - _Ask for a break appropriately_ |
| **_Connect_** | - _1:1 tasks_ - _Baseline of child’s behavior_ - _Regular check-ins to prompt regulation strategy_ - _Use simple, concise language_ - _Make empowering statements when distressed_ - _Build student’s self-esteem_   - _Non-contingent reinforcement_   - _Leadership roles_ | - _1:1 tasks_ - _Baseline of one’s feelings of child’s behavior_ - _Using gentle, specific language when giving demands_ - _Reinforce appropriate behavior_   - _Continuous positive attention_   - _Intermittent reinforcement_   - _Non-contingent reinforcement_ - _Lighten up the moment_ - _Collaborate with the student’s family_ | - _1:1 tasks_ - _Baseline of one’s feelings on child’s behavior_ - _Narrate your time with the student_ - _Narrate the student’s time with others_ - _Student leadership opportunities_ - _Collaborate with the student’s family_ |
| **_Cultivate_** | - _Use breaks as rewards_ - _Non-preferred activity for 5 minutes before a 20 minute break_ - _Rewards to reinforce use of self-soothing strategy_ - _Positive verbal reinforcement in the moment of anxiety to reinforce self-soothing_ - _Have student keep track of behaviors and strategies_ | - _Acknowledge only positive behavior_ - _Non-preferred activity for 5 minutes before a 20 minute break_ - _Validate the child’s feelings and then provide a choice_ - _Use incremental consequences_ - _Label the disagreement and encourage positive behavior_ - _Avoid prolonged discussions_ | - _Provide positive feedback_   - _Continuous positive attention_   - _Non-contingent reinforcement_ - _Avoid giving negative attention_   - _Reframe negative thoughts in the moment_   - _Neutral and quick response_ |
