# Supplementary material for: The Potential Emergence of “Education as Mental Health Therapy” as a Feasible Form of Teacher-Delivered Child Mental Health Care in a Low and Middle Income Country: A Mixed Methods Pragmatic Pilot Study
Source: Front Psychiatry. 2021 Dec 16;12:790536. doi: 10.3389/fpsyt.2021.790536 (PMC8717545; doi:10.3389/fpsyt.2021.790536)
Supplement: Supplementary file 5 [file Data_Sheet_5.docx]

**Supplementary Figure 5**. Teacher Time Log
