# Supplementary material for: The Potential Emergence of “Education as Mental Health Therapy” as a Feasible Form of Teacher-Delivered Child Mental Health Care in a Low and Middle Income Country: A Mixed Methods Pragmatic Pilot Study
Source: Front Psychiatry. 2021 Dec 16;12:790536. doi: 10.3389/fpsyt.2021.790536 (PMC8717545; doi:10.3389/fpsyt.2021.790536)
Supplement: Supplementary file 6 [file Data_Sheet_6.docx]

**Supplementary Figure 6**. Cause Analysis Chart (AABC) Evaluation Checklist

Evaluator: Date:

Teacher’s Name:________________________ Child’s Name: _______________________

| ***Domain*** | | ***Response*** | | | ***Comments*** |
| --- | --- | --- | --- | --- | --- |
| **Activating Event** | | **Yes (all)** | **Some (>50%)** | **No (50% or less)** |  |
| For each Behavior an Activating Event is identified. | |  |  |  |  |
| Each Activating Event is logically related to the triggered Behavior. | |  |  |  |  |
| Each Activating Event immediately precedes the triggered Behavior. | |  |  |  |  |
| Each Activating Event is clearly and specifically detailed and is not simply the setting or the activity. | |  |  |  |  |
|  | **Behavior** | | | | |
| The description provides a clear, specific picture of the Behavior. | |  |  |  |  |
| Each Behavior immediately precedes the Consequence. | |  |  |  |  |
| The Behavior is considered to be maladaptive. | |  |  |  |  |
|  | **Consequence** | | | | |
| For each Behavior a Consequence is identified. | |  |  |  |  |
| The Consequence provides a description of what happens directly after the Behavior. | |  |  |  |  |
| The Consequence is logically related to the Behavior. | |  |  |  |  |
|  | **Overall** | | | | |
| Number of observations completed is greater than or equal to 10. | |  |  |  |  |
| For each observation all supporting data (including date, time, duration, setting, and activity) are included. | |  |  |  |  |
| The chart demonstrates a clear use of knowledge and skills learned during training. | |  |  |  |  |
| Each observation is written with sufficient clarity and detail to allow others to understand it. | |  |  |  |  |
| The ABC Chart provides a good description of the activating event, behavior, and consequence. | |  |  |  |  |
| The ABC chart is completed based on direct observations completed by the teacher or obtained from family. | |  |  |  |  |

# **Supplementary Figure 6**. 4Cs Behavior Plan Evaluation Checklist

Evaluator: Date:

Teacher (Study ID):________________________ Child (Study ID): __________________

| ***Domain*** | ***Response*** | | ***Comments*** |
| --- | --- | --- | --- |
| **Cause** | **Yes** | **No**  **N/A** |  |
| One or more antecedent themes are identified. |  |  |  |
| One or more consequence themes are identified. |  |  |  |
| Demonstrates understanding of the concepts of antecedents and consequences. |  |  |  |
| Identified themes demonstrate a clear relationship between behavior and the antecedents & consequences |  |  |  |
| **Change** | | | |
| One or more school or home environmental changes are described. |  |  |  |
| Environmental changes relate logically to the problem behavior. |  |  |  |
| One or more organization or classroom learning strategy is described. |  |  |  |
| One or more new skills is identified. |  |  |  |
| Each identified skill has an accompanying strategy/action to promote acquisition. |  |  |  |
| The majority of proposed changes are based on the concept of positive behavioral support. |  |  |  |
| The majority of proposed changes are logistically feasible and attainable. |  |  |  |
| Inappropriate, shaming, or stigmatizing strategies are not included. |  |  |  |
| **Connect** | | | |
| One or more strategies for promoting social connection with the student in the classroom ins defined. |  |  |  |
| Strategies for creating classroom connections are clear, realistic, and attainable. |  |  |  |
| A clear, realistic, and attainable goal for one-on-one student engagement is defined. |  |  |  |
| There is a plan for one-on-one student engagement at least every other week/weekly. |  |  |  |
| **Cultivate** | | | |
| Includes two or more specific and actionable rewards for reinforcement of positive behaviors. |  |  |  |
| Includes two or more specific and actionable consequences to react to problem behavior and promote alternatives. |  |  |  |
| Identified rewards and consequences can be implemented over time to reinforce and maintain desired behaviors. |  |  |  |
| **Overall** | | | |
| Developmentally appropriate for the age of the student. |  |  |  |
| Demonstrates a clear use of skills and strategies learned during training. |  |  |  |
| Plan has been written with sufficient clarity and detail to allow others to understand and implement it. |  |  |  |
| Iterative changes occur over the course of the year as different parts of each “C” are trialed. |  |  |  |

**Additional Comments:**

1. What are some positives you observed on the 4Cs Plan?
2. What are some things this teacher can do to improve the 4Cs Plan?

# **Supplementary Figure 6**. One-on-one Student Interaction Evaluation Tool


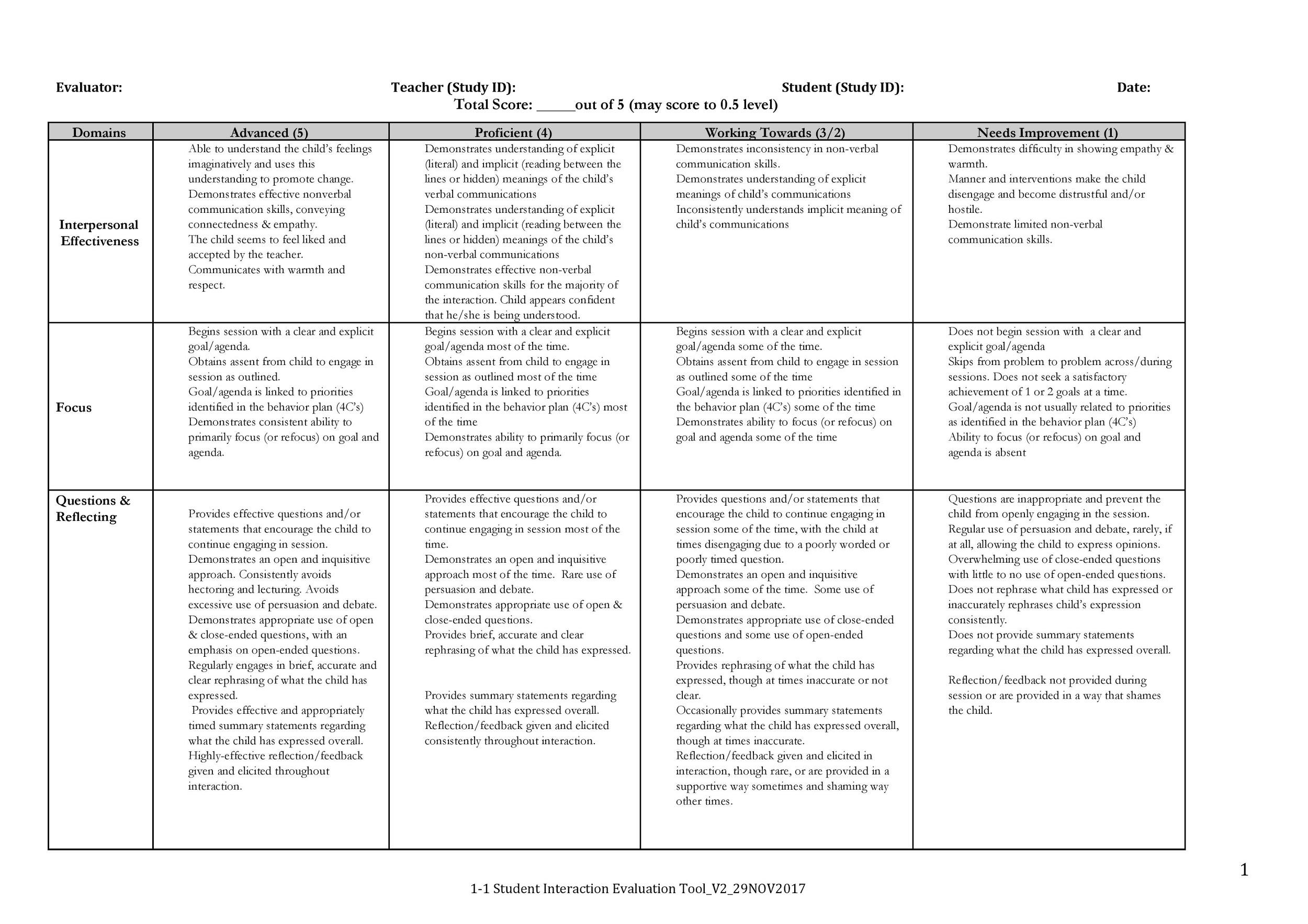


**
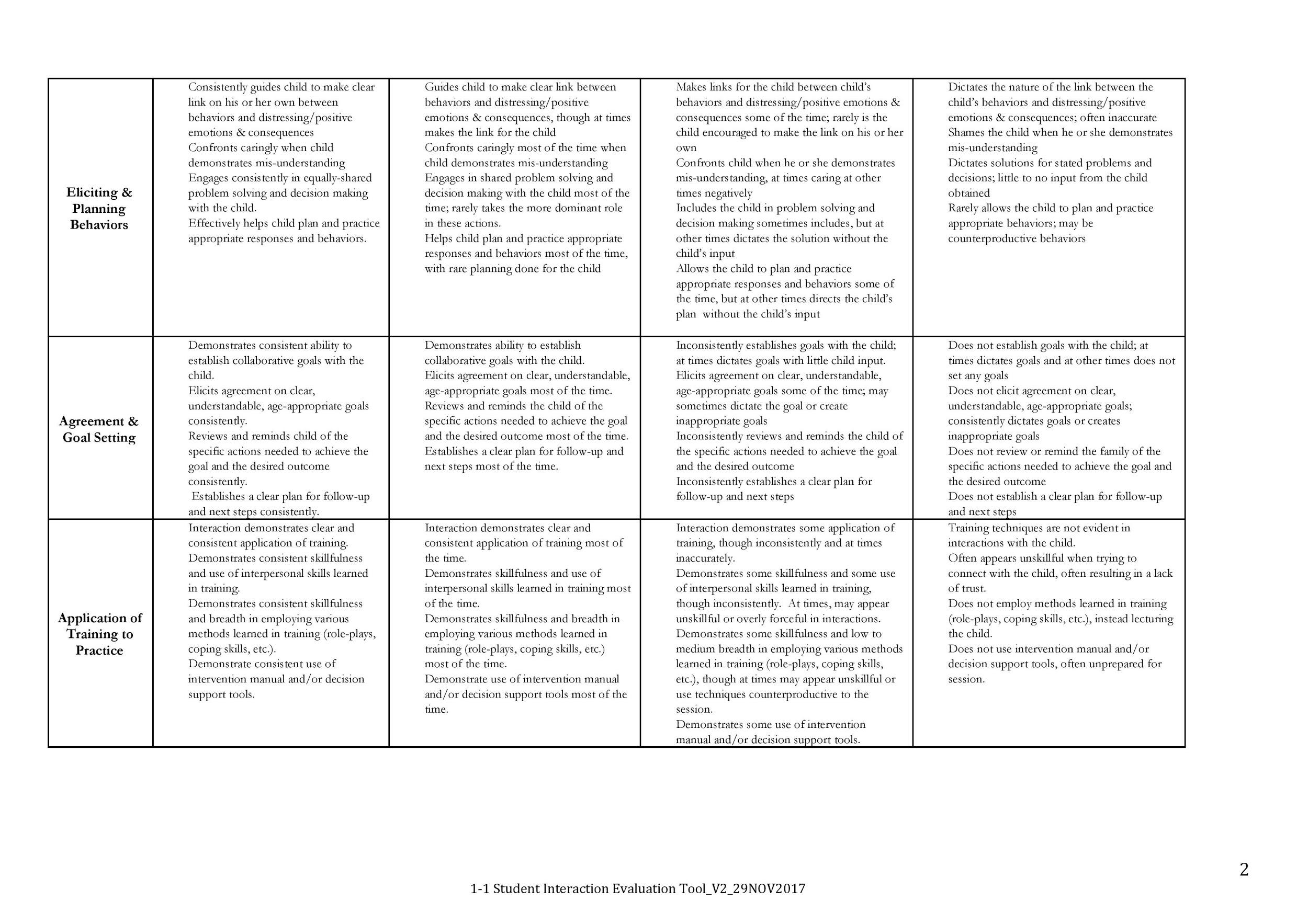
**

**
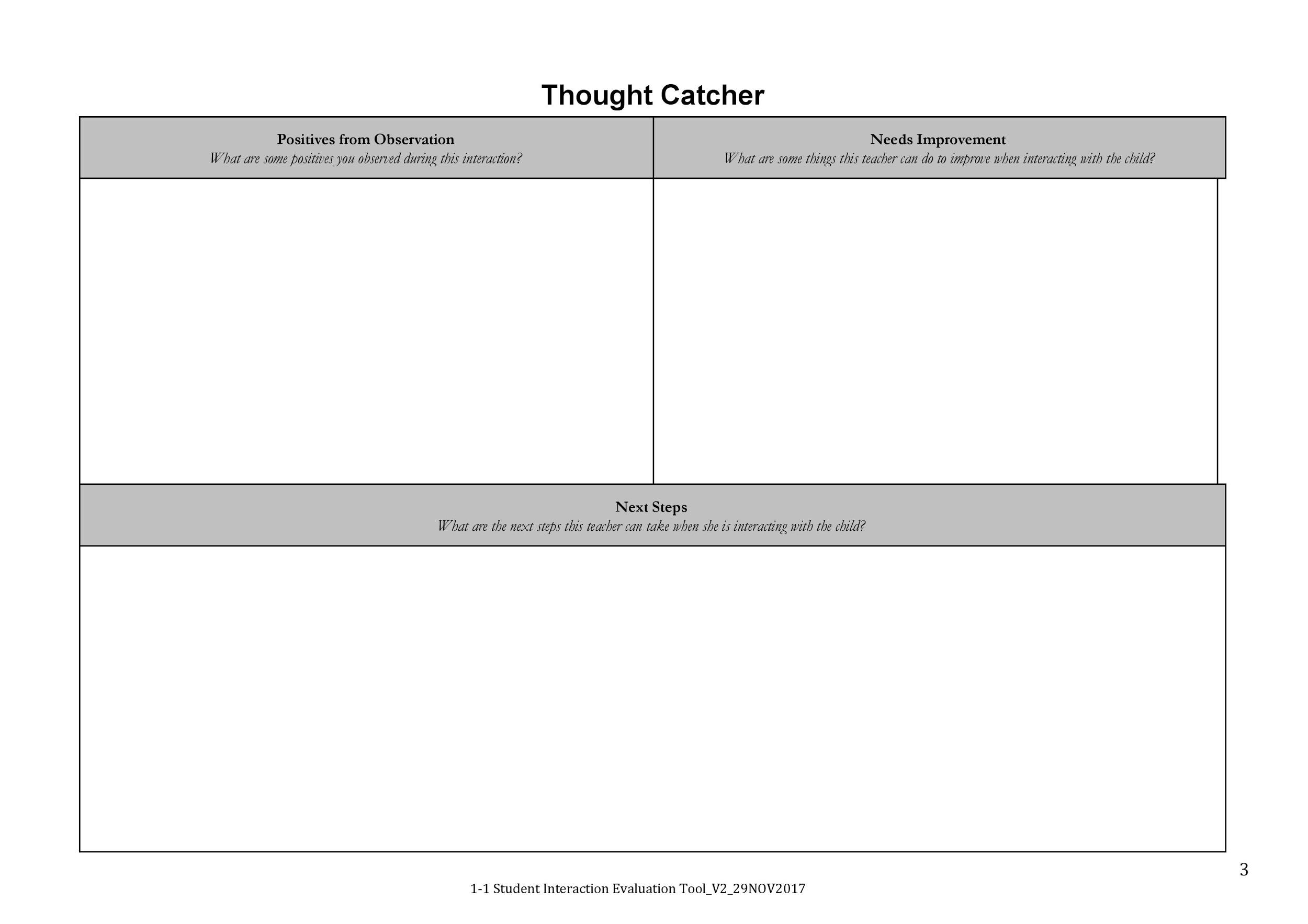
**

**Supplementary Figure 6**. One-on-one Family Interaction Evaluation Tool

**
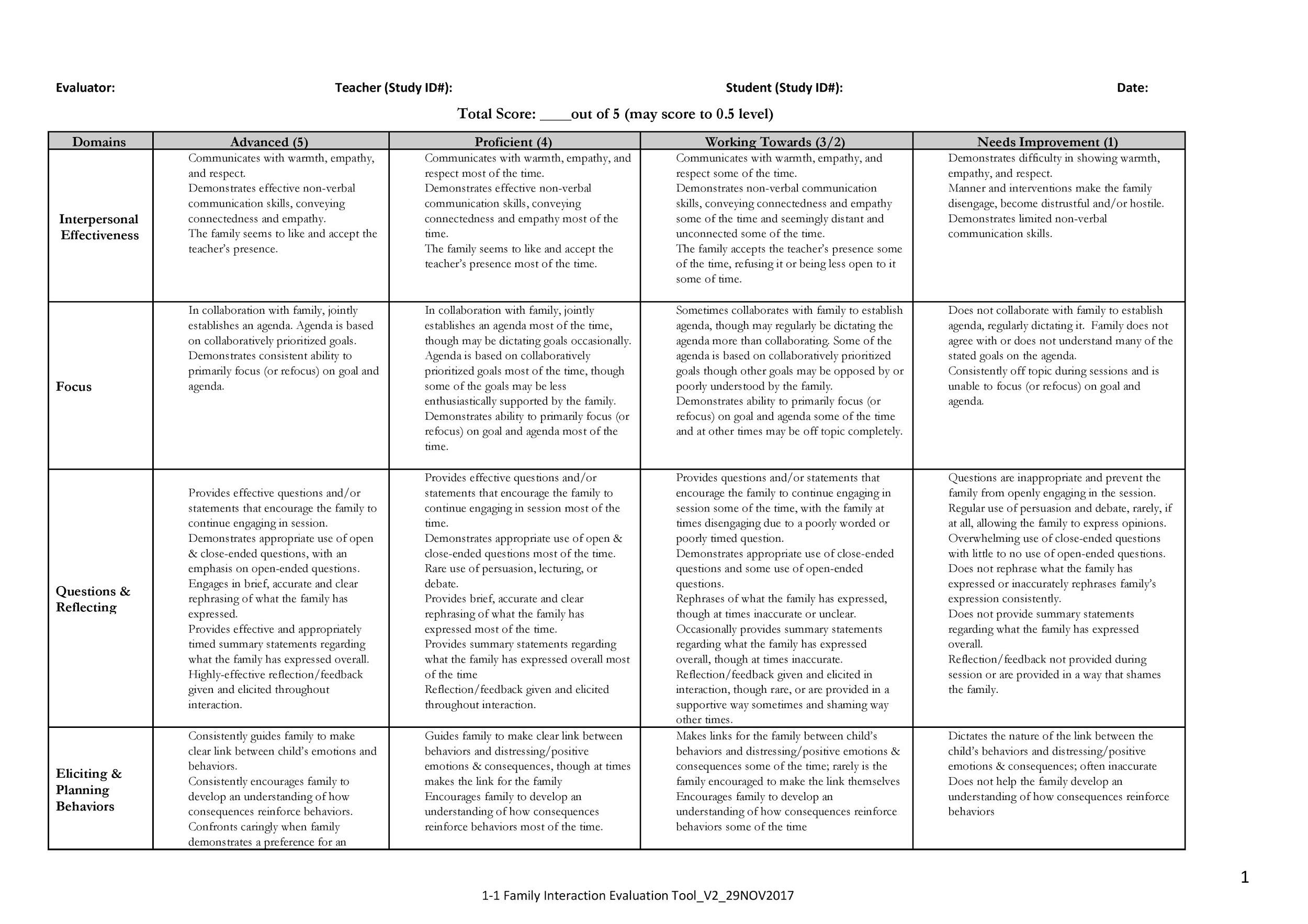
**


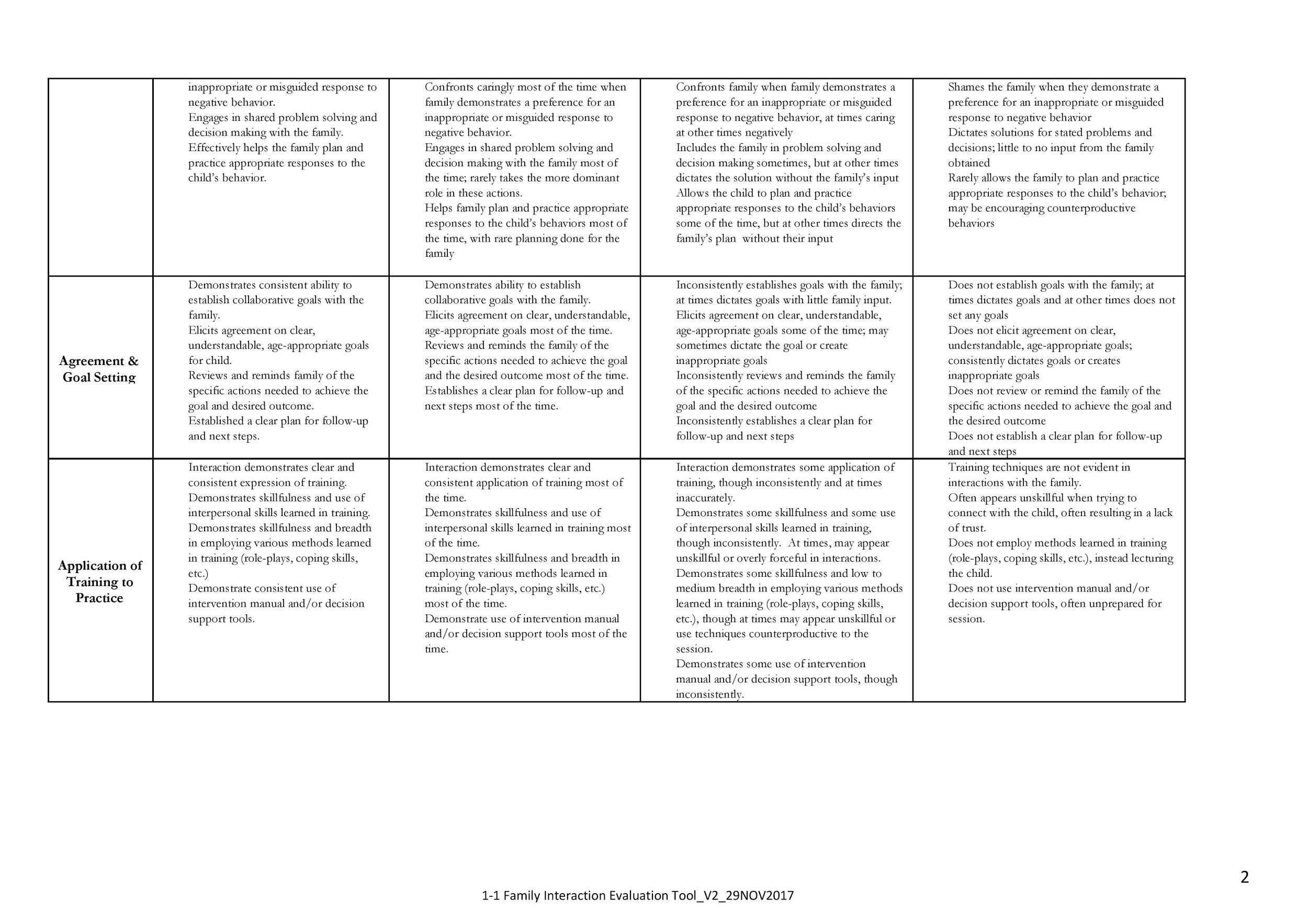
**
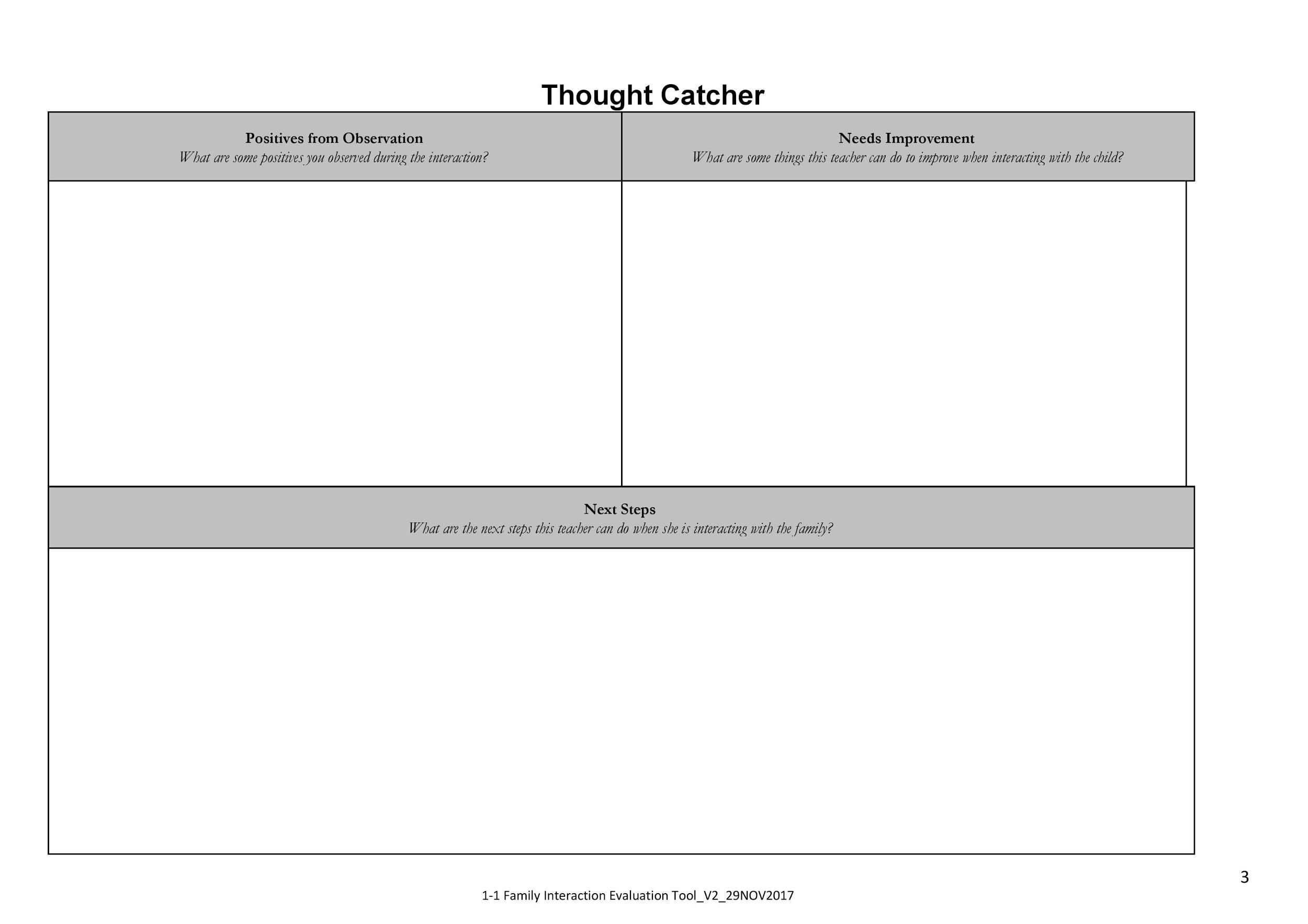
**
