# Supplementary material for: The Potential Emergence of “Education as Mental Health Therapy” as a Feasible Form of Teacher-Delivered Child Mental Health Care in a Low and Middle Income Country: A Mixed Methods Pragmatic Pilot Study
Source: Front Psychiatry. 2021 Dec 16;12:790536. doi: 10.3389/fpsyt.2021.790536 (PMC8717545; doi:10.3389/fpsyt.2021.790536)
Supplement: Supplementary file 7 [file Data_Sheet_7.docx]

# **Supplementary Figure 7**. Classroom Teachers Semi-Structured Interview Guide

***I am [name of research staff] from Darjeeling Prerna. Today we are conducting a participatory assessment of the child mental health program. This research will be used to help us understand the benefits and challenges of the program. We will use this information as part of our study to understand how the program worked and how we can improve it in the future.***

***As a reminder, your participation in this interview is voluntary. We can stop at any time. If any of the interview questions make you uncomfortable or upset, you can choose not to answer.***

***We will be audio recording this interview so that we can capture everything that you share with us. The tapes will be stored in our office in a locked cabinet and we will destroy them upon completion of the study. Everything that you share with us will be kept confidential.***

***We greatly appreciate you taking the time to share your thoughts and experiences with us!***

1. **Introduction**

***I’d like to start by asking you some general questions about the child metal health program***

1. Before the program started, did you feel that you needed additional training in how to manage and support children with challenging behaviors?
2. How much time per week do you think you spent completing the intervention? (*Probe: Was this enough time, not enough, too much?)*
3. Did you feel that the impact of the program (on you and your students) was worth the time and energy you devoted to it? (*Probe: Why/why not?)*

***Now I’d like to ask you about the specific components of the program***

1. **Intervention Components**
2. Training

***I will start with the training.***

1. After training did you feel ready and comfortable to recognize and support children with challenging behaviors? (*Probe: Why/why not?)*
2. What skills did you acquire during the training that you felt were most useful?

***I’d like to ask you some questions about the Behavior Analysis now.***

1. Behavior Analysis (ABC)
2. How long did it take you to complete the behavior observations and ABC charts? (*Probe: Did you feel that this was too long?)*
3. Did you complete a second (or third) ABC chart during your time working with the students? (*Probe: Why/why not?)*
4. Did completing the ABC chart shed light on your student’s challenging behavior? *(Probe: If so, how?)*

***I’d like to ask you some questions about the Behavior Plans.***

1. Behavior Plan (4C)
2. In general, how do you feel the behavior plan (4C plan) worked? *(Probe: What worked well? What did not work? What parts were you able to implement?)*
3. Did the 4Cs behavior plan change the way you interacted with your targeted student? (*Probe: If so, how? If not, why not?)*

***I ‘d like to ask you some questions about your individual interactions with the students.***

1. 1:1 Student Interaction
2. In general, how do you feel that counseling went with your students? (*Probes: What did you do that worked well? What did not work well?)*
3. What difficulties did you face when you were providing counseling (*Probe: Can anything be done about these difficulties?)*
4. When would you typically spend time working with the individual students (*Probes: Did you spend time with the students’ outside your normal workday? If yes, how did you feel about that?)*
5. When you were providing counseling to the students, can you tell me about specific tools or skills that you learned during training and found useful? (*Probes: Were there any skills or tools that you did not feel well prepared to use in “real-life”? Did you use any tools or strategies that we did not provide training on?)*

***I would like to ask you now about your interactions with the students’ families.***

1. 1:1 Family Interaction
2. In general, how do you feel that your interactions with the families went? (*Probes: What went well? What did not go well?)*
3. Was it difficult for you to find the time to interact with the family? (*Probes: When did you tend to interact with the family? Where did you generally meet with the family to work with them?)*
4. When you were working with the families what challenges did you face? (*Probes: How did you handle these challenges? Did you feel prepared to handle these challenges?)*
5. Do you feel like the parents trusted you to help them support their child? (*Probe: Why/why not?)*

***I’d like to ask you about the supervision and support you received from the project team.***

1. Supervision and Support
2. Tell us about the assistance that you received from the project team. (*Probes: What was the most helpful? Was there any feedback that you felt was unhelpful?)*
3. How often did your receive support from the project team? Was this enough? (*Probe: Ideally how often would you want to meet or discuss your work with the project team?)*

***Now I’d like to ask you about the results of the program***

1. **Overall**
2. Has the program changed you as a teacher? If so, how? (*Probe for specific examples)*
3. Did your students’ behaviors change as a result of the program and your work? If so, how? (*Probe for specific examples?)*
4. Did you observe any changes in the students’ academic performance? (*Probe for specific details)*
5. Did you observe any changes in how frequently the students were disciplined by our other members of the school staff? (*Probe for specific details)*
6. Did you acquire new skills that you plan to use in the future? If so, which skills?
7. Going forward would you be willing to continue to participate in the program (*Probe: Why/why not?)*

***And lastly,***

1. Do you have any other thoughts or suggestions for us regarding the program and how we can make it better in the future?

***Thank you for everything that you have done during this study and program. We greatly appreciate your participation! Please feel free to reach out to our team if you have any thoughts, questions, or concerns.***

***Thank you.***
