# Supplementary material for: The Potential Emergence of “Education as Mental Health Therapy” as a Feasible Form of Teacher-Delivered Child Mental Health Care in a Low and Middle Income Country: A Mixed Methods Pragmatic Pilot Study
Source: Front Psychiatry. 2021 Dec 16;12:790536. doi: 10.3389/fpsyt.2021.790536 (PMC8717545; doi:10.3389/fpsyt.2021.790536)
Supplement: Supplementary file 8 [file Data_Sheet_8.docx]

# **Supplementary Figure 8**. Mean (SE) TRF Total Problems Percentiles across the Intervention Time Period

Abbreviations: SE, standard error

Line graph demonstrating mean scores on the TRF total problem scale expressed as percentile scores. For scores above the 50th percentile, a decrease in scores indicates less deviance from normality (i.e. improved mental wellbeing). Per standard cut-offs, percentile scores less than 83^rd^ percentile are classified as normal, scores between 83-90^th^ percentile are considered borderline, and scores above the 90^th^ percentile are in the clinical range.
