# Supplementary material for: The Potential Emergence of “Education as Mental Health Therapy” as a Feasible Form of Teacher-Delivered Child Mental Health Care in a Low and Middle Income Country: A Mixed Methods Pragmatic Pilot Study
Source: Front Psychiatry. 2021 Dec 16;12:790536. doi: 10.3389/fpsyt.2021.790536 (PMC8717545; doi:10.3389/fpsyt.2021.790536)
Supplement: Supplementary file 9 [file Table_1.docx]

# **Supplementary Table 1**. Correlation between Fidelity and Child Mental Health

| **Correlation between Fidelity and Child Mental Health Outcomes** | | |
| --- | --- | --- |
| **Intervention Component** | **Pearson Correlation Coefficient** | **P Value** |
| Time | 0.46 | 0.009 |
| Behavior Analysis | -0.12 | 0.52 |
| Behavior Plan | -0.25 | 0.21 |
| 1:1 student interaction | 0.14 | 0.53 |
| 1:1 family interaction | -0.01 | 0.97 |

The results of a correlation analysis between a teacher’s fidelity scores and the associated change in TRF scores for their individual students are presented. Correlation is measured by Pearson correlation coefficient; results can range from -1 to 1 with values greater than 0 indicating a positive association.

Abbreviations: 1:1, one-on-one.
